# Flavin and deazaflavin biosynthesis in mycobacteria: relevance to physiology, implications for drug discovery, MR-1 antigenicity, and vaccine development

**DOI:** 10.3389/fimmu.2025.1656167

**Published:** 2026-01-16

**Authors:** Nurudeen Oketade, Melissa D. Chengalroyen, Dylan Kain, David M. Lewinsohn, Karen M. Dobos

**Affiliations:** 1 Department of Microbiology, Immunology and Pathology, Colorado State University, Fort Collins, CO, United States; 2 UCT Molecular Mycobacteriology Research, Institute of Infectious Disease and Molecular Medicine, Department of Pathology, University of Cape Town, Cape Town, South Africa; 3 Oregon Health and Science University, Portland, OR, United States; 4 Portland VA Medical Center, Portland, OR, United States

**Keywords:** flavin and deazaflavin, flavin sequestration, MR1T and MAIT cells, mycobacteria, therapeutics, tuberculosis, vaccines

## Abstract

Flavin and deazaflavin biosynthesis are highly conserved pathways in mycobacteria, including in *Mycobacterium tuberculosis* (*M.tb).* Flavin biosynthesis on one hand is required to produce FMN and FAD, two essential cofactors required to support the flavin intensive lifestyle of mycobacteria. Deazaflavin biosynthesis on the other hand provides F420, an important cofactor used by mycobacteria to curtail antimicrobial and immunological stressors. Given these crucial roles for mycobacterial survival and virulence, these connected pathways have been a recent focus of drug discovery efforts. In addition to providing these important cofactors, studies have shown that the intermediates of this pathway are required to produce metabolic antigens presented by the MHC class I related protein (MR1) molecule in mycobacteria. T cells restricted by the MR1 molecule, which includes Mucosal-associated invariant T cells (MAITs), have also been shown to play a key role during *M.tb* infection. These findings have made MR1 restricted T cells a prime target for vaccine development. In this review, we focus on what is known about flavin and deazaflavin synthesis pathways in *M.tb* and other mycobacteria and the distinct features in these species. We also cover the role of these pathways in the physiology of mycobacteria, as well as the status of small molecule inhibitors targeting this pathway. We discuss the current understanding of MR1 immunology in *M.tb* infection, based on studies in both animal models and humans. Additionally, we highlight recent findings on the diverse repertoire of MR1 T cell receptors that expand during infection and the current status of the MR1 ligandome. Most importantly, we discuss current gaps in understanding the importance of these pathways and explore how this knowledge could drive the development of therapeutics for mycobacterial diseases by targeting these pathways and protective MR1-restricted T cell responses.

## 1 Introduction

### 1.1 Mycobacterial diseases: a persistent global health challenge

The *Mycobacteria* genus comprises over 100 species, with approximately 30 recognized as pathogens causing a spectrum of infectious diseases in mammals (1–4). These pathogens are broadly categorized into three groups: the *M.tb* complex (MTBC), the *Mycobacterium leprae (M. leprae)* complex (MLC), and the nontuberculous mycobacteria (NTMs) (1, 3, 5). Together, these groups significantly contribute to the global human health burden, with *M.tb* and *M. leprae* being the most impactful. According to the World Health Organization (WHO), *M.tb* causes tuberculosis (TB)in approximately 11 million people and causes over 1 million deaths annually making it the deadliest infectious disease worldwide (6). *M. leprae*, though less common, still causes over 200,000 new cases of leprosy each year, a disease that leads to severe disability due to the bacterium’s ability to infect peripheral nerve cells (7). In addition to *M.tb* and *M. leprae*, NTMs are emerging as significant pathogens (8, 9). Species such as *Mycobacterium avium* (*M. avium*), and *Mycobacterium abscessus* (*M. abscessus*) are environmental opportunists (10) that primarily infect individuals with underlying conditions like cystic fibrosis (11, 12), bronchiectasis (13), chronic obstructive pulmonary disease (COPD) (14), or immunodeficiencies (15, 16). However, infections can also occur in immunocompetent individuals (17). The ubiquitous presence of NTMs in the environment makes them difficult to control, and their infections are often underreported due to diagnostic challenges and the absence of systematic global surveillance (10).

The global persistence of mycobacterial diseases is exacerbated by the rise in drug-resistant strains. For *M.tb*, multi-drug resistance (MDR) remains a significant concern, causing approximately 3% of new TB cases in 2023 (6, 18, 19). Similarly, *M. leprae* eradication efforts are hindered by the emergence of drug-resistant strains, with around 10% of cases resistant to at least one drug in the standard treatment regimen (20, 21). NTMs present an even greater challenge due to species-specific resistance patterns. For instance, *M. abscessus* demonstrates resistance to nearly all available antimycobacterial therapies (22–25). The intrinsic resistance mechanisms of NTMs, coupled with their ability to form biofilms (26, 27) that impede drug efficacy, make these infections particularly difficult to treat.

Vaccine development against mycobacterial diseases has seen little success in recent decades. The Bacillus Calmette-Guérin (BCG) vaccine, first introduced in 1921, remains the only approved vaccine for *M.tb (*28). While BCG provides protection against severe extrapulmonary and meningeal TB in children, it has limited efficacy against adult pulmonary TB, the most prevalent form of the disease (29). This highlights the urgent need for a more effective vaccine. For *M. leprae*, the absence of a conductive *in vitro* culture system has hindered the development of attenuated or killed vaccines (30). Similarly, the genetic and pathologic diversity of NTMs poses a barrier to creating a universal vaccine for this group of pathogens. Though BCG offers some cross-protection against leprosy (30) and NTM (31, 32) infections, its efficacy is limited.

The development of effective vaccines and novel antimycobacterial therapies is crucial to achieving the goals of the WHO's End TB Strategy (33) and the Global Leprosy Strategy (34). Addressing the drug resistance crisis and improving diagnostic tools for NTMs are equally important. Advancements in biotechnology, including genomic tools, high-throughput drug screening, and innovative vaccine platforms, hold promise for tackling the persistent challenge of mycobacterial diseases.

### 1.2 Flavin and deazaflavin biosynthesis as a target for drug discovery and vaccine development

Recent advances in antimycobacterial therapy have led to the approval of bedaquiline, delamanid, and pretomanid for TB treatment by the FDA (35, 36). Bedaquiline, which targets energy metabolism, has reignited interest in targeting bacterial metabolic pathways (37), particularly the central and essential secondary metabolic pathways in mycobacteria. This represents a paradigm shift, as most traditional antitubercular agents primarily target macromolecule synthesis. For instance, isoniazid and ethambutol inhibit the synthesis of the mycobacterial cell wall by targeting mycolic acid (38) and arabinogalactan/lipoarabinomannan (39) biosynthesis, respectively. Rifampicin and rifapentine disrupt transcription by inhibiting RNA polymerase (40), while fluoroquinolones such as moxifloxacin target DNA gyrase (41), an enzyme critical for DNA replication. Pyrazinamide acts by inhibiting the synthesis of coenzyme A, an essential molecule (42). Despite their success, these drugs face challenges such as resistance due to mutations and inactivity against non-replicating persisters (43–45). Consequently, the development of drugs targeting well-characterized and novel pathways remains a priority.

**Figure 1:**
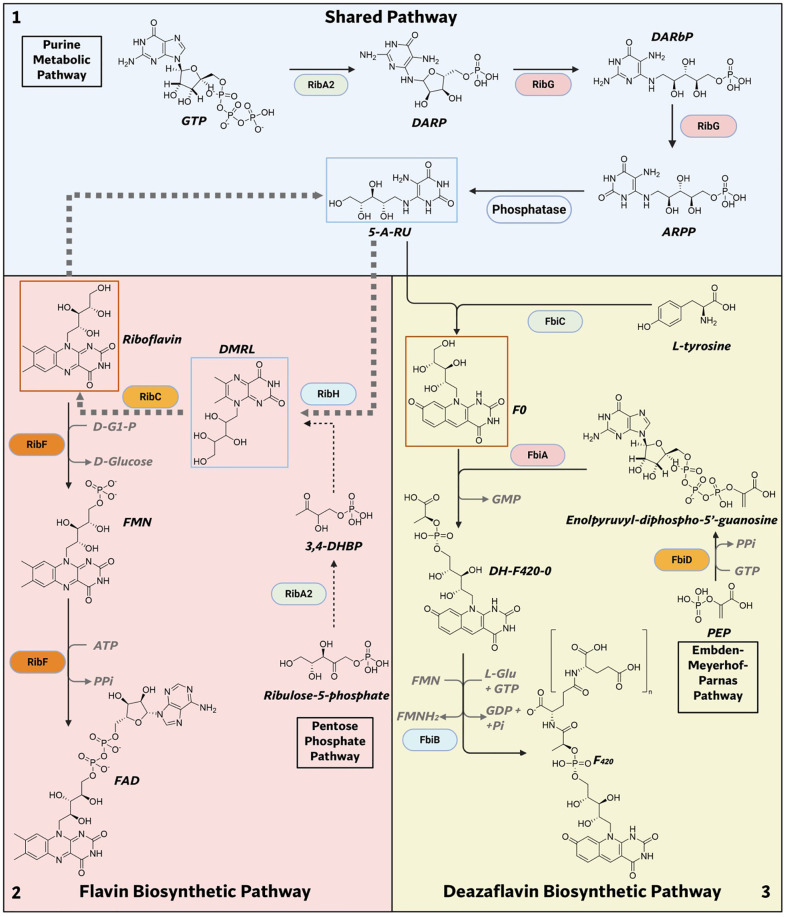
Summary of flavin and deazaflavin biosynthetic pathways in mycobacteria. Box 1: Shared pathway showing the synthesis of 5-A-RU. Box 2: Flavin biosynthetic pathway showing synthesis of FMN and FAD. Box 3: Deazaflavin biosynthetic pathway showing synthesis of F420. Blue boxed compounds: molecules known to serve as MR1 agonists or as their precursor. Orange boxed compounds: molecules known to bind MR1 without agonistic properties. (Image created with Biorender.com). GTP, guanosine triphosphate; DARP, 2,5-diamino-6-ribosyl-amino-4(3H)pyrimidinedione 5′-phosphate; DARbP, 2,5-diamino-6-ribityl-amino-4(3H)pyrimidinedione 5′-phosphate; ARPP, 5-amino-6-ribityl-amino-2,4(1H,3H)pyrimidinedione 5′-phosphate; 5-A-RU, 5-amino-6-D-ribitylaminouracil; 3,4-DHBP, 3,4-dihydroxy-2-butanone-4-phosphate; DMRL, 6,7-dimethyl-8-ribityllumazine; FMN, flavin mononucleotide; FAD, flavin adenine dinucleotide; PEP, Phosphoenolpyruvate.

One promising avenue is the flavin biosynthetic pathway (FBP) and the deazaflavin biosynthetic pathway (DBP), both of which are highly conserved in mycobacteria. The FBP synthesizes flavins, including flavin mononucleotide (FMN), and flavin adenine dinucleotide (FAD), which are metabolites that serve as indispensable cofactors for a wide range of enzymatic reactions (46, 47) (**Figure 1**). DBP synthesizes the deazaflavins 7,8-didemethyl-8-hydroxy-5-deazariboflavin (F0) and F420, metabolites that are involved in a variety of enzymatic reactions (48) (**Figure 1**). FMN and FAD are derived directly from riboflavin, a vitamin that humans obtain through diet due to the absence of the FBP in eukaryotes. These flavins are critical in mycobacteria because of their unique redox versatility, transitioning between fully oxidized (quinone), one-electron reduced (semiquinone), and two-electron reduced (hydroquinone) states (49). This property renders FMN and FAD irreplaceable by other redox cofactors, such as NAD and NADP (49). The flavin biosynthesis pathway is also upstream of another essential pathway, the vitamin B12 biosynthetic pathway. The reduced form of FMN is used to produce 5,6-dimethylbenzimidazole (DMB) (50), which is the lower axial ligand of vitamin B12, which in turn is a cofactor for several enzymes in *M.tb*. F0 serves as the precursor for F420, an obligate two-electron carrying deazaflavin essential for oxidative homeostasis in mycobacteria (48). F420 is also necessary for activating the antibiotics delamanid and pretomanid (51), further underscoring its therapeutic importance. Unlike FMN and FAD, which serve as essential cofactors in human metabolism, F0 and F420 and their dependent enzymes are absent in humans (52). The essentiality of FMN, and FAD (46, 53), coupled with the role of F420 in surviving oxidative stress during infection (54, 55), positions the FBP and DBP as quintessential drug targets. Additionally, the absence of both pathways in mammals minimizes the risk of off-target effects, making it an attractive avenue for drug discovery.

Beyond its metabolic significance, flavin biosynthesis produces specific intermediates presented by the non-classical presenting molecule MHC Class I-Related Protein (MR1) (56, 57). These metabolites are recognized by MR1-restricted T cells (MR1T cells), a unique subset of donor-unrestricted T cells (DURTs). Unlike conventional T cells that recognize peptide antigens, MR1T cells respond to FBP and DBP intermediates through semi-invariant T-cell receptors (TCRs) (57). These MR1T cells can be further subclassified into Mucosal Associated Invariant T (MAIT) cells, which are defined by their expression of the semi-invariant alpha-chain TRAV1-2, and non-TRAV1–2 expressing MR1T cells, which have a more diverse TCR repertoire (58, 59). MAIT cells have been shown to reside in mucosal surfaces (60, 61), including the respiratory tract, the primary infection site of *M.tb* and several NTMs. Emerging evidence suggests that MAIT cells play a crucial role in controlling mycobacterial infections (62–66) and represent exciting targets for novel therapeutics and vaccines against mycobacteria (67). However, advancing this field requires prioritizing the identification and characterization of the antigenic metabolic produced by mycobacteria.

Historically, the FBP garnered significant attention during the mid-20th century due to its essentiality in bacterial survival (68–74). However, the discovery of alternative drug targets and antimicrobials led to a decline in interest. Moreover, limitations in biotechnological tools at the time hindered deeper exploration of this pathway. With recent technological advances, such as genome editing, high-throughput drug screening, structural biology, and metabolomics, the potential of the FBP as a drug target can now be fully realized. These tools will enable precise investigations into the metabolic capabilities of mycobacteria and the therapeutic exploitation of the FBP.

In this review, we aim to comprehensively describe flavin and deazaflavin biosynthesis and metabolism in mycobacteria, emphasizing its potential as a target for drug discovery and vaccine development. We will highlight existing research gaps that need to be addressed to harness this pathway for therapeutic innovation. Additionally, we will discuss how modern biotechnological tools can accelerate the exploration and exploitation of this vital metabolic pathway.

## 2 Overview of the flavin and deazaflavin biosynthetic pathway

The study of flavin biosynthesis in mycobacteria dates to the early 1900s, inspired by the striking yellow pigment observed in cultures of *M.tb* and extracts from *M. leprae (*68, 70). This observation led to efforts to isolate and characterize the pigment, which was later identified as riboflavin (68). The identification of riboflavin marked a significant milestone in understanding the metabolic capabilities of mycobacteria. Subsequent research explored the relationship between riboflavin production and the environmental conditions under which mycobacteria were grown (69). Investigators sought to determine whether variations in growth conditions influenced riboflavin levels and whether exogenous riboflavin could enhance the growth of the bacilli (69). While these studies provided foundational insights, they yielded inconclusive results regarding the physiological role of riboflavin in mycobacteria. Another avenue of early research focused on the potential connection between riboflavin biosynthesis and virulence. Some studies hypothesized that riboflavin production might contribute to the pathogenicity of mycobacteria, while others examined whether riboflavin deficiency in infected hosts played a role in disease progression (68, 73). Despite these intriguing hypotheses, the limitations of early experimental techniques meant that these questions remained largely unanswered. Although many of these investigations were inconclusive, they laid the groundwork for future research. With the advent of advanced biotechnological tools such as CRISPR-based gene editing, transcriptomics, proteomics, metabolomics, and mass spectrometry imaging, it is now possible to revisit these early questions with greater precision. Modern approaches have started to provide critical insights into the role of riboflavin and deazaflavin biosynthesis in mycobacteria and its potential connections to virulence, host-pathogen interactions, and metabolic adaptability. Revisiting these early studies in the context of contemporary science could uncover novel aspects of mycobacteria biology with implications for therapeutic strategies.

The discovery that mycobacteria could produce F0 and F420 was made relatively recently (48, 75). The initial discovery of F0 and F420 biosynthesis was made in archaeal methanogens (76). However, due to the estranged nature of the archaeal genome compared to other domains, no connection was made to the synthesis of F0 and F420 in bacteria (77, 78). In the early 1980s, F0 was isolated from *M. avium (*79) and later discovered in other members of the mycobacterial genus (48). Interestingly, the evolutionary origin of F0 and F420 was eventually tied to *Actinobacteria (*80), a phylum to which the mycobacteria genus belongs. The evolutionary conservation in this phylum therefore highlights the physiological importance of these compounds. Although the biosynthesis of F0 and F420 is not essential for viability (81), evidence suggests that they play a role in redox homeostasis and detoxification of environmental stressors in mycobacteria (54, 82–84).

Genetic manipulation experiments including gene knockdown, gene knockout, and complementation studies have provided insight into the importance of these pathways (46, 54, 83). Additionally, since the discovery of these pathways in mycobacteria, it has also become apparent that the final product of this pathway is not limited to the currently known catalog of molecules (85–87). In the next section, we cover the enzymes required for flavin and deazaflavin biosynthesis, the genetic architecture of the pathway, its uniqueness in comparison to other flavin and deazaflavin-producing microorganisms, and production of tangential metabolites.

### 2.1 Core enzymes and reactions

**Figure 2:**
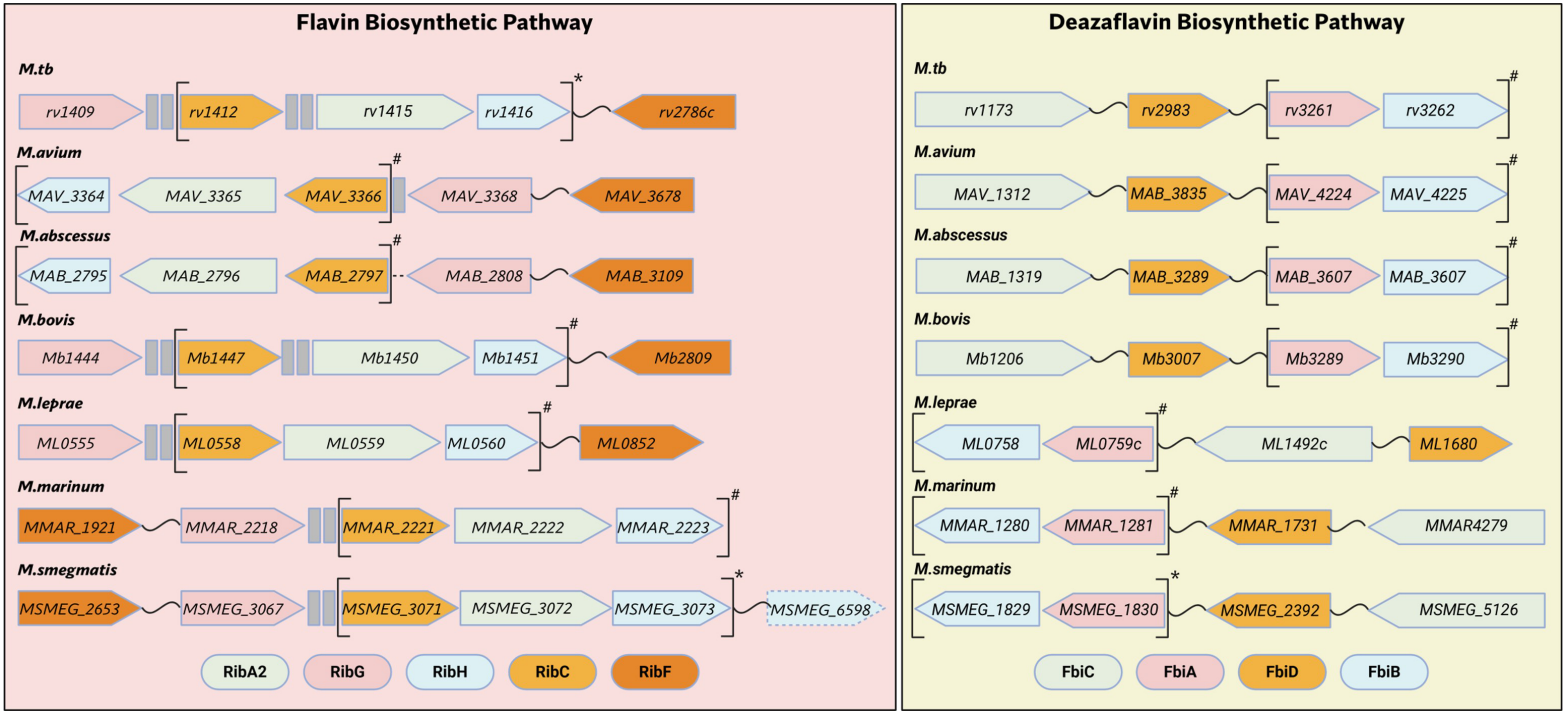
Genetic architecture of flavin and deazaflavin biosynthetic genes in mycobacteria. Genes are color coded according to enzymes in **Figure 1**. Genes in brackets are experimentally confirmed (*) or hypothesized (#) to be operonic. (Image created with Biorender.com).

The FBP and DBP together consist of nine enzymes (**Figures 1**, **2**). Three of these enzymes (RibA2, RibG and an uncharacterized phosphatase) are shared between the two routes (88–90). These shared enzymes are required for the conversion of guanosine triphosphate (GTP) to 5-Amino-6-(ribityl-amino) uracil (5-A-RU) (88). Subsequently, 5-A-RU is converted into riboflavin by the two enzymes, RibH and RibC and eventually FMN and FAD by RibF (47). Alternatively, for the synthesis of F0 and F420, four enzymes are required, namely FbiA, FbiB, FbiC and FbiD to which 5-A-RU serves as the starting material (91–93). Here, we go over the core enzymes of this pathway and the similarity and differences to well characterized organisms such as *Bacillus subtilis (B. subtilis)* and *Escherichia coli (E. coli)*. Biochemical characterization of these pathways is beyond the scope of this review, but has recently been reviewed elsewhere (94, 95).

#### 2.1.1 FBP and DBP shared pathway

The first step in flavin biosynthesis is catalyzed by the bifunctional RibA2 enzyme (GTP cyclohydrolase II/DHBP synthase). RibA2 catalyzes the hydrolytic release of a carbon and pyrophosphate from GTP to form 5-amino-6-ribosylyamino-4(3H)-pyrimidinone 5`-phopshate (47). RibG, another bifunctional enzyme, firstly deaminates the pyrimidine ring and then reduces the ribosyl side chain of the product of RibA2 to form 5-amino-6-ribitylamino-2,4(1H,3H)-pyrimidinedione 5`-phosphate (47). The product of RibG is then dephosphorylated to form 5-amino-6-D-ribitylaminouracil (5-A-RU). The phosphatase responsible for the dephosphorylation step is unknown but it is hypothesized to be catalyzed by a phosphatase with low substrate specificity (96). 5-A-RU then serves as the common substrate for the synthesis of both flavins and deazaflavins.

#### 2.1.2 Riboflavin biosynthesis

For flavin synthesis, the first step involves the condensation of 5-A-RU with 3,4-dihydroxy-2-butanone 4-phosphate (3,4-DHBP) by RibH, a lumazine synthase, to form 6,7-dimethyl-8-ribityllumazine (DMRL). 3,4-DHBP is formed from ribulose 5-phosphate by RibA2 via a dismutase reaction (97). Although, the condensation of 5-A-RU and 3,4-DHBP to form DMRL can occur non-enzymatically in an aqueous solvent, it has been shown that RibH is required for DMRL synthesis in mycobacteria (46). In the final step, RibC (riboflavin synthase) condenses two molecules of DMRL to form one molecule of riboflavin and one molecule of 5-A-RU (88). RibF, a bifunctional enzyme, first phosphorylates riboflavin to form FMN which it then converts to FAD (88).

#### 2.1.3 Deazaflavin biosynthesis

The synthesis of F0 involves the condensation of 5-A-RU with tyrosine. This reaction is catalyzed by a two-domain fusion protein FbiC (F0 synthase) (98). The synthesis of F420 from F0 requires both phosphoenolpyruvate (PEP) and GTP. PEP is guanylylated by FbiD (93), a guanylyltransferase to form enolpyruvyl-diphospho-5′-guanosine (EPPG). The transferase FbiA then transfers PEP to F0 to form dehydro F-420-0 (91). FbiB, a two-domain protein, reduces dehydro-F-420–0 via its reductase domain to afford F-420-0 (91). An oligoglutamate tail is then added to F-420–0 by FbiB via its γ-glutamyl ligase domain (99, 100). The oligoglutamate tail has been shown to contain between five to seven glutamate residues in mycobacteria (48).

### 2.2 Genetic architecture and uniqueness of the FBP in mycobacteria

The FBP and DBP are highly conserved across Mycobacteria, as illustrated in **Figure 2**. In *M.tb* and *Mycolicibacterium smegmatis (M.smeg)*, three key genes of this pathway—*ribC*, *ribA2*, and *ribH*—are organized in an operon known as the *rib* operon (46). The *ribG* gene is located separately from the operon with two intervening open reading frames (ORFs) and appears to have no transcriptional relationship with the *rib* operon (46). Additionally, *ribF* is expressed as a standalone gene, situated distally from the other pathway genes. In some mycobacterial species, two ORFs that are not involved in riboflavin biosynthesis are found embedded within the *rib* operon. The high degree of synteny in the arrangement of these operonic genes across different mycobacterial species suggests that they may also be expressed as an operon in other members of this genus. This operonic arrangement of riboflavin biosynthesis genes are a common feature in eubacteria. For example, in *B. subtilis*, all the genes of the riboflavin biosynthetic pathway (RBP) are clustered in a single operon regulated by a FMN riboswitch (101). The FMN riboswitch serves as a negative feedback regulatory mechanism to prevent the excess production of riboflavin due to the high energy cost of this process and to ensure redox homeostasis (102). A similar mechanism exists in *E. coli*, despite the genes being scattered across its genome (102). Bioinformatic studies have suggested that mycobacteria lacks a FMN riboswitch regulating the RBP (103), which may indicate that there are other mechanism(s) in place to mitigate the problem of excess riboflavin. One such mechanism could be flavin sequestering proteins that is highly conserved in mycobacteria, which will be discussed later in this review (104, 105). Another reported mechanism is the redox homeostatic system (RHOCS) made up of protein kinase G (PknG), ribosomal protein L13 and RenU, a Nudix hydrolase. The disruption of the RHOCS prevents the degradation of FAD and NAD(P)H by RenU, leading to their accumulation (106). However, the RHOCS is not specific for alleviating redox stress due to flavins as this system primarily senses high levels of NADH. It is also probable that the basal rate of riboflavin biosynthesis provides the right quantity of cofactors (FMN and FAD) needed to drive the high flavin dependence of mycobacteria (107).

A common feature of the RBP in bacteria is the presence of redundant systems to ensure sufficient riboflavin supply. These mechanisms include duplicate pathway genes, which may afford protection from inhibitory molecules targeting riboflavin biosynthesis (108), as well as the ability of some pathogenic bacteria to also encode a riboflavin uptake mechanism (109). The presence of redundant supplies of riboflavin has been shown to be important in the colonization of the host by certain pathogens with the dependence on exogenous or endogenous source of riboflavin varying based on environmental conditions (108). Bioinformatic annotation of the *M.tb* genome indicate the presence of two *ribA* genes, *rv1940* and *rv1415*, as well as a second putative deaminase, *rv2671 (*110). However, functional studies confirmed that only *rv1415* encodes a functional enzyme (46), and that *rv2671* encodes a dihydrofolate reductase (DHFR) rather than a deaminase (111), thus indicating the presence of a sole gene for all the steps of this pathway. Conversely, in the non-pathogenic *M.smeg*, a redundant gene encoding a lumazine synthase (RibH) was observed (46). In terms of riboflavin uptake mechanisms, of the nine different families of riboflavin importers, none has been bioinformatically observed in mycobacteria (112).

The lack of redundancy of riboflavin supply and dependence on a sole ORF for each step of riboflavin biosynthesis in pathogenic mycobacteria suggests that targeting this pathway for therapeutics should be relatively straightforward. Additionally, distinct features of the mycobacterial RBP could be leveraged to develop targeted therapeutics that selectively inhibit mycobacteria while sparing the host microbiome. One major concern with antimicrobial therapies is the unintended disruption of commensal bacteria, which can have significant health consequences. However, structural and functional differences in the mycobacterial RBP compared to other bacteria present an opportunity to design highly specific inhibitors. In some riboflavin-competent organisms, the function of hydrolyzing the GTP ring and synthesizing 3,4-dihydroxy-2-butanone-4-phosphate (DHBP) is carried out by two separate enzymes, RibA and RibB (108, 113), whereas in mycobacteria, this process is consolidated into a single multifunctional enzyme (114). These distinctions could serve as a basis for designing inhibitors that exploit the structural and mechanistic uniqueness of mycobacterial riboflavin biosynthesis while avoiding off-target effects on beneficial microbiota which is a strategy currently employed by bedaquiline, an antimicrobial that specifically targets the mycobacterial ATP synthase. By targeting these species-specific variations in flavin biosynthesis, it may be possible to develop antimycobacterial agents that effectively combat infections without the collateral damage associated with broad-spectrum antibiotics. This approach underscores the importance of detailed biochemical and structural characterization of the mycobacterial RBP for the development of therapeutics.

Given the essentiality of flavin biosynthesis and the flavin intense lifestyle of mycobacteria, it is intriguing that pathogenic mycobacterial species do not encode redundant mechanisms to ensure the supply of these vital molecules (46, 107, 115). This phenomenon can likely be attributed to the absence of evolutionary pressure to develop redundancy. Pathogenic mycobacteria colonize traditionally sterile anatomical sites, such as the lungs or peripheral nerves, where they encounter minimal microbial competition. This sterility reduces the likelihood of exposure to inhibitory molecules or metabolic competition from other bacteria, thereby diminishing the selective pressure to evolve backup systems for flavin production. By contrast, environmental mycobacteria and other bacteria that coexist in competitive ecosystems are often subjected to such pressures, which may drive the evolution of metabolic redundancy or alternative pathways to ensure survival (10). For example, photolumazines, which have only been observed in *M.smeg*, can serve as inhibitors of riboflavin synthase which may provide an advantage in the midst of other environmental microorganisms (116). This distinction emphasizes the unique metabolic adaptations of pathogenic mycobacteria, shaped by their specialized niches within the host, and further highlights flavin biosynthesis as a vulnerable and attractive target for therapeutic development.

### 2.3 Genetic architecture and uniqueness of the DBP in mycobacteria

Interestingly, the initial genome annotation of *M.tb* in 1998 did not include genes required for deazaflavin biosynthesis (110). In 2001, *fbiA* and *fbiB* were identified as essential for F420 biosynthesis in *Mycobacterium bovis* (*M. bovis*) through PA-824 (now pretomanid)-induced selection of transposon mutants (117). The same group later identified *fbiC* using a similar approach (92), and the discovery of *fbiD* followed, linked to mutations in its ORF that conferred resistance to pretomanid and delamanid (93). Like the *rib* operon, two of the *fbi* genes, *fbiA* and *fbiB*, are juxtaposed and have been shown to be co-transcribed (118), while *fbiC* and *fbiD* are located at separate loci (**Figure 2**). Intriguingly, the genetic architecture of the deazaflavin pathway in mycobacteria differs from the archaeal pathway. Five genes instead of four are required for F420 synthesis in archaebacteria with the role of *fbiC* requiring two separate genes *cofG* and *cofH (*89). Also, the length of the polyglutamate tail of F420 has been shown to be shorter in the archaeal organisms in comparison to mycobacteria (90, 119). The distinct mechanisms through which different organisms are able to regulate the length of the glutamate tail is yet to be elucidated. The length of the polyglutamate tail has been shown to impact the kinetics, the turnover rate, and the interaction of F420 with oxidoreductases (120). Shorter tail length has also been linked to resistance (121), however, whether mycobacteria can vary the tail length depending on environmental conditions has yet to be fully understood.

Initial studies characterizing deazaflavin biosynthesis had shown that L-Lactyl-2-diphospho-5`-guanosine (LPPG), a metabolite made from the guanylylation of 2-Phospho-L-lactate via FbiD, was required for the synthesis of F420 (122, 123). However, a study by Bashiri et al. showed that PEP rather than LPPG was required for F420 biosynthesis in mycobacteria (99). This clarification of the substrate of FbiD was due to the lack of genetic and biochemical evidence supporting the synthesis of 2-Phospho-L-lactate in mycobacteria, while PEP was a well characterized product of the glycolytic pathway. This discovery challenged the long-standing schema of this pathway. Recently, the diverse nature of this pathway has become more obvious, with different organisms using molecules other than PEP for F0 synthesis (89). For example, the Gram-negative bacterium *Paraburkholderia rhizoxinica* utilizes 3-phopsho-D-glycerate (3PG) instead of LPPG or PEP (124). The use of PEP rather that LPPG in mycobacteria was further supported by the presence of a FMN binding domain in FbiC which catalyzes the reduction of dehydro-F420-0 (99), the product of FbiA if PEP is used. Also, the synthesis of F0 was thought to require the condensation of 5-A-RU and 4-hydroxyphenylpyruvate (4-HPP). However, the biochemical characterization of FbiC enabled better elucidation of F0 synthesis and demonstrated the need for tyrosine instead of 4-HPP.

### 2.4 Tangential pathways beyond the FBP and DBP

Beyond the production of flavins and deazaflavins, 5-A-RU may participate in the synthesis of additional metabolites. Notably, 5-A-RU condenses non-enzymatically with glyoxal and methylglyoxal, secondary products of metabolism, to form 5-(2-oxoethylideneamino)-6-D-ribitylaminouracil (5-OE-RU) and 5-(2-oxopropylideneamino)-6-D-ribitylaminouracil (5-OP-RU) (125). These unstable metabolites are potent ligands for MR1. Additionally, other studies have demonstrated the formation of lumazine compounds through the non-enzymatic condensation of 5-A-RU with transamination products (126, 127). The enzymatic modification of 5-A-RU to provide starting material for a tangential metabolic pathway has also been observed in other organisms (128). The promiscuity of 5-A-RU and the non-enzymatic production of its derivatives suggest that this pathway may contribute to the synthesis of other metabolic products. Although most known products of 5-A-RU outside flavins and deazaflavins have only been characterized as MR1 antigens, it is necessary to determine whether these metabolites have other physiological roles in mycobacteria. In addition, the distinct metabolome of mycobacteria (129, 130) compared to other organisms may provide an avenue for the formation of novel secondary metabolites derived from 5-A-RU.

Another point in the FBP that may lead to the synthesis of other novel metabolites is from DMRL. DMRL, the direct precursor for riboflavin, belongs to the broad class of nitrogen-containing heterocycles known as pteridines or lumazines. The ribityl lumazine motif serves as a core structure for several naturally occurring compounds in various bacterial species, often carrying diverse additional moieties that contribute to their biological functions. One notable class, photolumazines, has been shown to be produced by *M.smeg (*85, 86), and is yet to be observed in any pathogenic species. Photolumazines may play a role in protecting *M.smeg* in light-exposed environments as these compounds function in association with lumazine binding proteins as optical transponders (131). Therefore, the presence of photolumazines and their functional role in pathogenic mycobacteria such as *M.tb* and *M. leprae* is likely minimal or absent, given their evolution to survive in the absence of light. However, NTMs, often sourced from environmental niches (10), may produce photolumazines. Since both DMRL and 5-A-RU have been implicated in the formation of lumazines in other organisms, it is critical to investigate pathogenic mycobacteria for the presence of such substrates and to explore their potential physiological and immunological relevance.

FBP also provides a precursor essential for cobalamin biosynthesis. Specifically, reduced flavin mononucleotide (FMN-H_2_) undergoes reduction by BluB, a nitroreductase-like enzyme, yielding DMB and erythrose-4-phosphate (E4P) (50). Subsequently, DMB is incorporated into cobalamin through additional enzymatic reactions (50). While cobalamin is critical for mycobacterial metabolism, members of the MTBC typically obtain it exogenously from their host due to an incomplete *de novo* synthesis pathway (132, 133). In contrast, NTMs possess the complete machinery to synthesize cobalamin independently (132), relying on DMB derived from FMN. The broader implications of disrupting the synthesis of cobalamin lie beyond the scope of this review but have been previously addressed elsewhere (134).

With the wealth of information now available about these pathways and the expanding repertoire of therapeutic strategies beyond traditional small-molecule inhibitors, there is significant potential to develop novel antimycobacterial agents. Additionally, the conservation of flavin and deazaflavin biosynthesis among mycobacteria suggests that inhibitors designed to target this pathway in one species will likely be effective against others. For example, bedaquilline targets ATP synthase, a highly conserved function in mycobacteria, and has demonstrated potent antibacterial activity against several mycobacterial species (135–137). This broad applicability enhances the feasibility of developing a single therapeutic agent capable of targeting multiple pathogenic mycobacteria, making flavin and deazaflavin biosynthesis an attractive target for the next generation of antimycobacterial drugs.

## 3 Flavin biosynthesis as a target for drug discovery

At the time of the discovery of riboflavin production in mycobacteria, there was significant interest in identifying metabolites essential for the growth of pathogens (74). The prevailing assumption was that structural analogs of these metabolites could serve as starting points for antimicrobial development, an approach inspired by the success of prontosil, a dihydropteroate synthase inhibitor (138, 139), as an antibacterial agent. However, because riboflavin is utilized by both prokaryotic and eukaryotic organisms, the strategy of modifying riboflavin for antimicrobial purposes had to be approached with caution. While the absence of the flavin and deazaflavin biosynthetic pathway in humans makes its intermediate steps promising targets for drug discovery, no successful clinical antimicrobial agents have been developed to disrupt this pathway to date.

**Figure 3:**
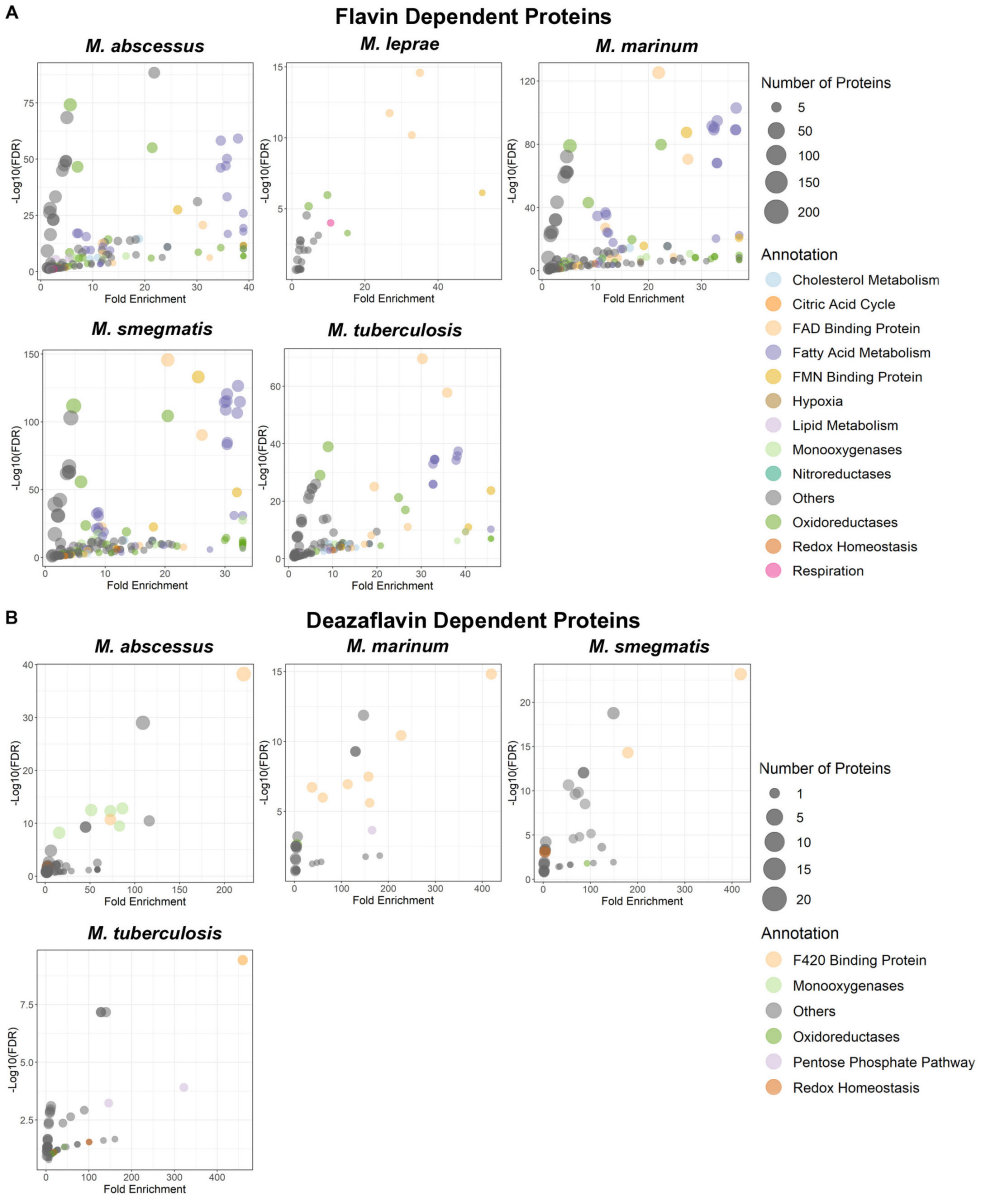
Gene ontology enrichment analysis for flavin and deazaflavin dependent proteins in mycobacteria. Flavin-dependent **(A)** and deazaflavin-dependent **(B)** proteins in mycobacterial species were identified from the UniProtKB database based on their annotation as an FMN, FAD or F420 dependent/binding protein. Gene Ontology (GO) enrichment analysis was conducted with ShinyGO (v0.82) (154), using all available proteins pulled from UniProtKB as described. Statistical significance was determined using a false discovery rate (FDR) threshold of 0.05 to correct for multiple hypothesis testing. Fold enrichment was calculated as the ratio of observed to expected gene counts in each GO term, indicating the degree of overrepresentation. Enriched GO terms were manually reviewed and grouped into broader functional categories to facilitate interpretation of biological themes.

The supply of riboflavin, essential for the synthesis of FMN and FAD, is indispensable for both eukaryotic and prokaryotic organisms (140). The ability of the alloxazine ring to maintain a quinone, semi-quinone and fully reduced state makes its utility as a cofactor unique (49). In mycobacteria, the extensive presence of flavoproteins suggests a flavin-intensive metabolic lifestyle (107). The functional roles of these flavoproteins span critical cellular processes such as fatty acid metabolism, cholesterol metabolism, nucleotide biosynthesis, and redox homeostasis (**Figure 3**). Major components of the electron transport chain (ETC) either contain flavoproteins or directly utilize FAD/FMN as cofactors. These include NADH dehydrogenase-1 (NDH-1) (141), a multi-subunit enzyme complex, NDH-2 (142), a single-polypeptide enzyme, and succinate dehydrogenase (SDH) (143).

F420, although dispensable for viability, plays a crucial role in mycobacterial physiology (89, 99). The loss of F420 biosynthesis may not alter viability but has been shown to negatively impact the fitness of mycobacteria (75, 82–84, 144), indicating the lack of compensatory mechanisms for this loss. Unlike FMN and FAD, F420 is an obligate two-electron carrier with a low redox potential similar to NAD(P)H (89, 90). Its precursor, F0, appears to serve solely as an intermediate in F420 biosynthesis in mycobacteria. Genes required for F420 synthesis have been identified in most clinically relevant mycobacteria. Of particular interest is the conservation of F420 biosynthesis in *M. leprae (*48), a species that has undergone extreme genome reduction. This suggests that F420 is functionally significant despite the minimal genetic repertoire of *M. leprae*. Additionally, bioinformatic analyses have revealed numerous F420-dependent genes in mycobacteria (82, 145), and biochemical characterization has confirmed the F420 dependency of several protein products (146–148). These enzymes participate in key physiological processes, including cell wall biosynthesis (148), respiration (149), redox homeostasis (84, 149, 150), and the degradation or inactivation of antimicrobials and intoxicants (151, 152). Some of these functions have been inferred from genetic disruption of F420 biosynthesis (83, 93, 117, 148, 152), yet the primary proteins responsible for these roles remain unidentified. Identifying these primary players will provide deeper insight into their role in the virulence of pathogenic mycobacteria and inform the development of strategies to directly inhibit or circumvent these mechanisms, thereby enhancing both immunological and antimicrobial clearance.

Although the catalog of characterized flavoproteins and deazaflavoproteins in mycobacteria has expanded in recent years (**Figure 3**), there remains a significant gap in our understanding of the role of these groups of proteins. The *M.tb* genome is predicted to encode approximately 150 flavoproteins and 33 deazaflavoproteins with similar numbers expected in other mycobacterial species (82, 107). However, only a subset of these flavoproteins and deazaflavoproteins have been experimentally characterized. Comprehensive characterization of these proteins and their functional roles is essential for understanding some of the core metabolic processes required for mycobacterial survival. With advancements in bioinformatics and biochemical affinity techniques such as click chemistry, we now have the tools to facilitate this characterization. Since both riboflavin and F0 can be taken up passively (46, 90), these approaches offer a promising path toward uncovering novel therapeutic targets in mycobacteria.

### 3.1 Physiological roles of flavins and deazaflavins in mycobacteria

There are currently over 200 families of enzymes that depend on FMN or FAD as cofactors (153) and 8 families that depend on deazaflavins (90). These proteins are involved in a plethora of functions in nature and in the mycobacteria species. We have highlighted some of these functions in mycobacteria in **Figure 3**.

In this section, we provide an overview of the function of key flavin dependent enzymes, flavoproteome, and deazaflavin dependent enzymes in the context of features essential to the pathogenicity of mycobacteria.

#### 3.1.1 Energy metabolism/respiration

ATP production is essential for the viability of mycobacteria (155–157), and to ensure a continuous supply of energy under diverse environmental and chemical stressors, the bacterium has evolved a robust ATP-generating system based on oxidative phosphorylation. While some components of the ETC are functionally redundant and can be substituted, others are indispensable (158–160). Key entry points into the ETC (161),including the NDH-1, NDH-2, and SDH, require FAD as a cofactor. The essentiality of the two NADH dehydrogenases is underscored by the inability to generate a double mutant lacking both enzymes (162). Given the flavin dependence of these critical enzymes, disruption of flavin biosynthesis is expected to impair ETC function and compromise ATP production. Additional ETC-linked enzymes such as malate:quinone oxidoreductase (Mqo) (163, 164), proline dehydrogenase (Pru) (165, 166), and lipoamide dehydrogenase (Lpd) (167, 168), also require flavin cofactors, further underscoring the centrality of flavin metabolism. Consequently, even alternative electron entry routes that might compensate for the loss of NDH function are likely to be non-functional in the absence of flavins.

In addition to canonical flavin-dependent pathways, mycobacteria may utilize deazaflavin, F420, as an electron carrier. The deazaflavin-dependent nitroreductase (Ddn) (149), a peripheral membrane protein known for activating the prodrug pretomanid (169), has been shown to reduce menaquinone and is required under hypoxic conditions. The genome of *M.tb* encodes two additional homologs, Rv1261c and Rv1558, which exhibit enzymatic activities similar to Ddn and may similarly contribute to menaquinone reduction (149). However, unlike the flavin-dependent ETC components, the roles of these F420-dependent quinone reductases in electron transport remain to be fully elucidated and warrant further investigation.

#### 3.1.2 Redox homeostasis

Redox homeostasis is the ability of living systems to maintain a balance between oxidative and reductive species, ensuring a non-toxic intracellular environment. Since fundamental biological processes inherently impact an organism's redox status, redox homeostasis is essential for survival. This is particularly critical for pathogenic mycobacteria, which encounter oxidative and nitrosative stress in the form of reactive oxygen species (ROS) and reactive nitrogen species (RNS) within the macrophage phagosome during infection. To counteract these host-imposed stressors, mycobacteria have evolved multiple strategies, including the use of flavin and deazaflavin redox couples (FAD/FADH_2_, FMN/FMNH_2_, and F420/F420-H_2_) (89, 170).

FMN and FAD serve as cofactors for various proteins involved in redox homeostasis. A prime example is their role in recycling thiol-based redox buffers in mycobacteria (171). Mycothiol (MSH), a low-molecular-weight thiol unique to mycobacteria, functions as a redox buffer by neutralizing oxidative stress (172). During this process, MSH is oxidized to mycothiol disulfide (mycothione, MSSM), concomitantly reducing ROS. The flavoprotein mycothiol disulfide reductase (Mdr) catalyzes the conversion of MSSM back to its reduced form, MSH, restoring the redox buffer capacity (170). Loss of Mdr impairs bacterial growth and increases sensitivity to oxidative stressors, whereas its overexpression enhances resistance to oxidative stress. Another flavoprotein, ThyX, a thymidylate synthase (173), has also been implicated in oxidative stress protection in mycobacteria (174), although the precise mechanism is yet to be elucidated. Lipoamide dehydrogenase, Lpd, a flavoprotein is part of the peroxynitrite reductase/peroxidase (PNR/P) complex, a system involved in the detoxification of RNIs during infection (175). TyzC, a flavin-dependent oxidase (FDO) of the nitroreductase (NTR) superfamily has also been implicated in redox homeostasis as a transposon mutant of *tyzC* exhibited increased sensitivity to oxidative stress (176).

The deazaflavin cofactor F420 also plays a crucial role in mycobacterial redox homeostasis. Initial studies demonstrated that F420-deficient mutants exhibit heightened sensitivity to oxidative stress, later attributed to the requirement of reduced F420 (F420-H_2_) for oxidative stress resistance (83, 149, 150). The reduction of F420 is catalyzed by an F420-dependent glucose-6-phosphate dehydrogenase (Fgd), which links glucose-6-phosphate (G6P) metabolism to oxidative stress responses (146, 150). The loss of Fgd results in increased sensitivity to oxidative stress, further underscoring its importance in redox homeostasis (177). Interestingly, *M. leprae* encodes Fgd as its sole glucose-6-phosphate dehydrogenase (150), although whether it plays a similar role in redox homeostasis in this species remains unknown. Fgd is also present in other mycobacteria, including NTMs (150), but its contribution to redox homeostasis across species requires further investigation. Interestingly, the preference for Fgd over the more common NADP-dependent glucose-6-phosphate dehydrogenase in mycobacteria under oxidative stress conditions has not been elucidated. Understanding this preferential utilization could provide deeper insights into how mycobacteria orchestrate their metabolic and redox responses during host infection.

#### 3.1.3 Central carbon metabolism

Central carbon metabolism in mycobacteria plays a vital role in energy generation and the provision of precursors required for the synthesis of essential macromolecules such as DNA, RNA, lipids, and amino acids. The core central carbon metabolism network consists of the Embden–Meyerhof–Parnas pathway, the pentose phosphate pathway (PPP), and the tricarboxylic acid cycle (TCA) (178). Additionally, mycobacteria encode supplementary pathways such as the glyoxylate shunt and the methylcitrate cycle (178, 179), which enable metabolic adaptation under nutrient-limited or hostile conditions. While the bacilli is capable of extensive remodeling of these pathways by switching entirely to supplementary routes or bifurcating metabolic flux depending on environmental and nutritional cues, certain core enzymatic functions remain essential (179, 180). Several of these enzymes require flavin or deazaflavin cofactors for their activity. SDH (181), which bridges the TCA cycle and the ETC, catalyzes the oxidation of succinate to fumarate using FAD as an electron carrier. Similarly, Lpd (168), a flavoprotein within the pyruvate dehydrogenase complex of the EMP pathway, facilitates the conversion of pyruvate to acetyl-CoA, a central intermediate of the TCA cycle (182). While Fgd is primarily linked to redox homeostasis through the generation of reduced F420-H_2_ (177), its activity may also influence central carbon flux. Specifically, the upregulation of Fgd during oxidative stress suggests a potential shift in metabolism favoring the PPP, which is a major source of NADPH for detoxification processes. However, the mechanistic basis for this metabolic reprogramming and its regulation in response to oxidative cues remains to be fully elucidated.

Lactate serves as an alternative carbon source for mycobacteria, particularly within the host environment, where infected macrophages shift their metabolism toward aerobic glycolysis (183). This host metabolic reprogramming results in elevated lactate levels, which mycobacteria can exploit. The bacilli encodes two lactate dehydrogenases, LldD1 and LldD2 (184). Of these, LldD2, a flavin-dependent enzyme, is functionally active in oxidizing lactate to pyruvate (184). The resulting pyruvate can enter the TCA cycle or serve as a substrate for gluconeogenesis via a phosphoenolpyruvate carboxykinase (PckA)-dependent pathway (178). Importantly, LldD2 is essential for intracellular survival, as its deletion leads to impaired growth within macrophages (183). Furthermore, LldD2 has been identified as a target of evolutionary pressure; mutations within its ORF have been associated with increased expression (184), highlighting its role as a key metabolic enzyme and potential driver of virulence in mycobacteria.

#### 3.1.4 Fatty acid and cholesterol metabolism

The metabolism of fatty acids (FAs) and cholesterol is a cornerstone of mycobacterial survival and immunomodulation during infection (185, 186). Within macrophages, the bacterium efficiently utilizes host-derived fatty acids and cholesterol as primary carbon sources (186). In addition to catabolizing these lipids, mycobacteria synthesizes a variety of lipid species and lipid-containing molecules that contribute to the unique physicochemical properties of its cell wall and function as virulence factors through interactions with host immune components (187, 188). Flavoproteins and deazaflavoproteins are essential to both the catabolism of host lipids and the anabolism of cell wall-associated fatty acids. The β-oxidation of fatty acids and cholesterol yields acetyl-CoA and propionyl-CoA, which feed into the TCA cycle and gluconeogenesis, supporting both energy production and biosynthetic needs (186). Mycobacteria encode a large number of FAD-dependent acyl-CoA dehydrogenases (ACADs) involved in the β-oxidation of fatty acids (189). These enzymes function in concert with the electron transfer flavoprotein (ETF) system, composed of FixA, FixB, EtfA, EtfB, and EtfD (189, 190). Among these, EtfA and EtfB are flavoproteins that mediate the transfer of electrons from ACADs to the menaquinone pool in the ETC (189). For cholesterol degradation, mycobacteria express several flavin-dependent dehydrogenases, including ChsE3, HsaA, HsaB, KshB, LpdC, FadE30, LpdA, and LpdB and an F420-dependent oxidoreductase (Rv3520c). These enzymes play critical roles in the sequential breakdown of the cholesterol side chain and ring structures, facilitating the assimilation of cholesterol-derived carbon (186).

Flavins and deazaflavins also play a role in the biosynthesis of fatty acids and complex lipids that constitute the lipid-rich cell wall of mycobacteria. The bacterium encodes a multifunctional type I fatty acid synthase (FAS-I), responsible for the *de novo* synthesis of fatty acids ranging from C_16/C18_ to C_24/C26_ (191). These products are either incorporated into basic membrane phospholipids or funneled into the type II fatty acid synthase (FAS-II) system for the synthesis of mycolic acids, essential components of the mycobacterial cell envelope (192). The enoyl-ACP reductase component of FAS-I, which catalyzes the final and rate-limiting step of fatty acid chain elongation, is FMN-dependent (191), making it a key flavoprotein in lipid biosynthesis. Several complex lipids with defined roles in virulence also require flavin or deazaflavin cofactors for their synthesis. Phthiocerol dimycocerosates (PDIMs) are apolar lipids located in the outermost layer of the mycobacterial cell wall and function as major virulence factors by promoting phagosomal escape and inhibition of autophagy (193, 194). The biosynthesis of PDIM depends on phthiodiolone ketoreductase (fPKR), an F420 H_2_-dependent enzyme (195). Mutants deficient in PDIM synthesis are significantly attenuated in virulence, demonstrating the importance of this pathway in immune evasion (193, 194). Similarly, ketomycolic acids, which contribute to pellicle formation and drug tolerance, are synthesized from hydroxymycolic acids via an F420-dependent dehydrogenase (196). More recently, the production of acyl-tyrazolone (acyl-Tyz), a tyrosine-derived lipid, has been observed in mycobacteria (197). This compound is synthesized by a flavin-dependent nitroreductase-like enzyme, although its precise function and relevance to virulence are still under investigation (197). Collectively, these findings underscore the essential role of flavoproteins and deazaflavoproteins in the biosynthesis of key lipid-based virulence determinants in mycobacteria. Targeting flavin biosynthesis may not only compromise cell wall integrity but also increase cell wall permeability, potentially enhancing the efficacy of other antimicrobials that are otherwise impeded by the bacteria’s lipid-rich barrier.

#### 3.1.5 Drug resistance/detoxification

The detoxification of xenobiotic compounds significantly enhances the survival and resilience of mycobacteria. To neutralize antimicrobial agents, mycobacteria utilize specialized enzymes such as nitroreductases and monooxygenases. The flavin-dependent monooxygenase MabtetX in *M. abscessus* efficiently inactivates tetracycline and doxycycline, illustrating a critical drug resistance mechanism dependent on flavins (198). Another example is the flavin-dependent nitroreductase NfnB, conferring resistance to BTZ-043 in *M. smeg (*199). Although direct homologs of MabtetX and NfnB have not been identified in other mycobacterial species, their existence strongly suggests analogous mechanisms elsewhere within the genus.

Additionally, mycobacteria employ deazaflavin-dependent detoxification pathways. The loss of cofactor F420 synthesis has been correlated with increased susceptibility to various antimycobacterial drugs (200). This enhanced sensitivity is primarily due to the essential role of F420-H_2_ in enabling reductases to catalyze the detoxification of these antimicrobials (201). Specifically, several F420-dependent reductases from *M. smeg* are capable of reducing and inactivating toxic compounds (200, 201). Further illustrating this mechanism is the deazaflavin-dependent quinone reductase Ddn, known for activating the prodrug pretomanid (169). Interestingly, Ddn and its homologs such as Rv1261 and Rv1558 have been proposed to also participate in antimicrobial detoxification processes (149). The apparent functional redundancy of Ddn in species like *M.tb* and *M. avium (*202), which encode multiple homologs, underscores the robustness of these detoxification mechanisms.

Genome-wide bioinformatic analyses have identified more than 30 distinct flavin/deazaflavin-dependent oxidoreductase (FDOR) homologs within individual mycobacterial species (202). Although directly testing the relationship between flavin-dependent pathways and drug resistance is challenging due to their essential biological roles, pharmacologically targeting flavin biosynthesis may nonetheless enhance mycobacterial sensitivity to existing antimicrobial treatments.

#### 3.1.6 Dormancy/persistence

The success of *M.tb* and other mycobacteria as pathogens lies in its remarkable ability to persist under intense immunological and antimicrobial pressure. To achieve this persistent state, the bacterium undergoes a profound reorganization of its metabolism, shifting its focus from replication to survival. This metabolic shift is accompanied by a change in drug susceptibility, rendering the bacillus tolerant to multiple antimycobacterial agents (203). One of the major stressors encountered during infection is the reduction in oxygen tension within granulomas (204–207), organized immune structures formed to contain the infection. In response to this hypoxic environment, mycobacteria initiates a transcriptional adaptation orchestrated by the DosRST two-component regulatory system (208). DosS and DosT act as histidine sensor kinases, while DosR serves as the response regulator (209). These kinases sense environmental cues such as nitric oxide (NO), carbon monoxide (CO), and hydrogen sulfide (H_2_S) (209). Upon sensing these gases, DosS and DosT become activated and phosphorylate DosR, which then triggers the upregulation of genes within the Dos regulon. This regulon, comprising approximately 14 to 50 genes depending on the mycobacterial species, includes nitroreductases, ferredoxins, heat shock proteins, diacylglycerol acyltransferases, universal stress proteins, alternative electron transport components, and elements essential for anaerobic respiration (210). Among the two sensor kinases, DosS plays a particularly critical role in sustaining regulon activation and has been classified as flavin-dependent (211). Its activation is linked to its function as a redox sensor, with FMN proposed to participate in signal transduction. However, the exact mechanism through which flavin mediates DosS activation remains incompletely understood. Studies have shown that disruption of the Dos regulon attenuates *M.tb*, with deletion of *dosS* causing a more pronounced loss of virulence compared to deletion of *dosR* or *dosT*. This suggests that DosS may have functions beyond the classical regulon, potentially coordinating broader responses to environmental stress. Another key player in the dormancy response is Acg (*acr* co-regulated gene), also known as Rv2032, which is a potential flavin-sequestering protein (212). Acg is part of the Dos regulon and is strongly upregulated under hypoxic conditions (210). Its specific function remains under investigation, but its regulation and structure implicate it as a significant factor in the bacterium’s survival strategy during dormancy which will be discussed further in this review.

In addition to the flavin- and deazaflavin-dependent components discussed, mycobacteria encodes numerous other flavoproteins including several nitroreductases and monooxygenases (82, 107) that contribute to its metabolic versatility and stress adaptation. Collectively, these observations underscore the critical role of flavins and deazaflavin in maintaining mycobacterial physiology. Disruption of flavin and deazaflavin biosynthesis would likely have pleiotropic effects, impairing multiple essential pathways highlighted above. This vulnerability is particularly relevant in the context of rising resistance to current antitubercular regimens. Furthermore, the likelihood of resistance evolving through mutations in the flavin biosynthetic pathway may be limited, given that such mutations could impose significant fitness costs on the bacterium.

### 3.2 Inhibitors of flavin biosynthesis in mycobacteria

The flavin biosynthetic pathway presents several druggable targets, as five known enzymes within this pathway (RibA2, RibG, RibH, RibC, and RibF) are essential for bacterial viability. Among these, only RibF, a bifunctional riboflavin kinase/FAD synthase, has a homolog in the human host. Since humans are incapable of synthesizing riboflavin, they rely on dietary intake. This riboflavin is subsequently converted to FMN and FAD by separate enzymes, a riboflavin kinase and FAD synthase respectively, whereas both of these functions are carried out by the RibF enzyme in prokaryotes (213). The conservation of the riboflavin kinase domain of RibF between prokaryotes and eukaryotes in both sequence and structure makes it a less druggable target, however the FAD synthase domain displays significant structural divergence which may serve as a drug target (214).

**Figure 4:**
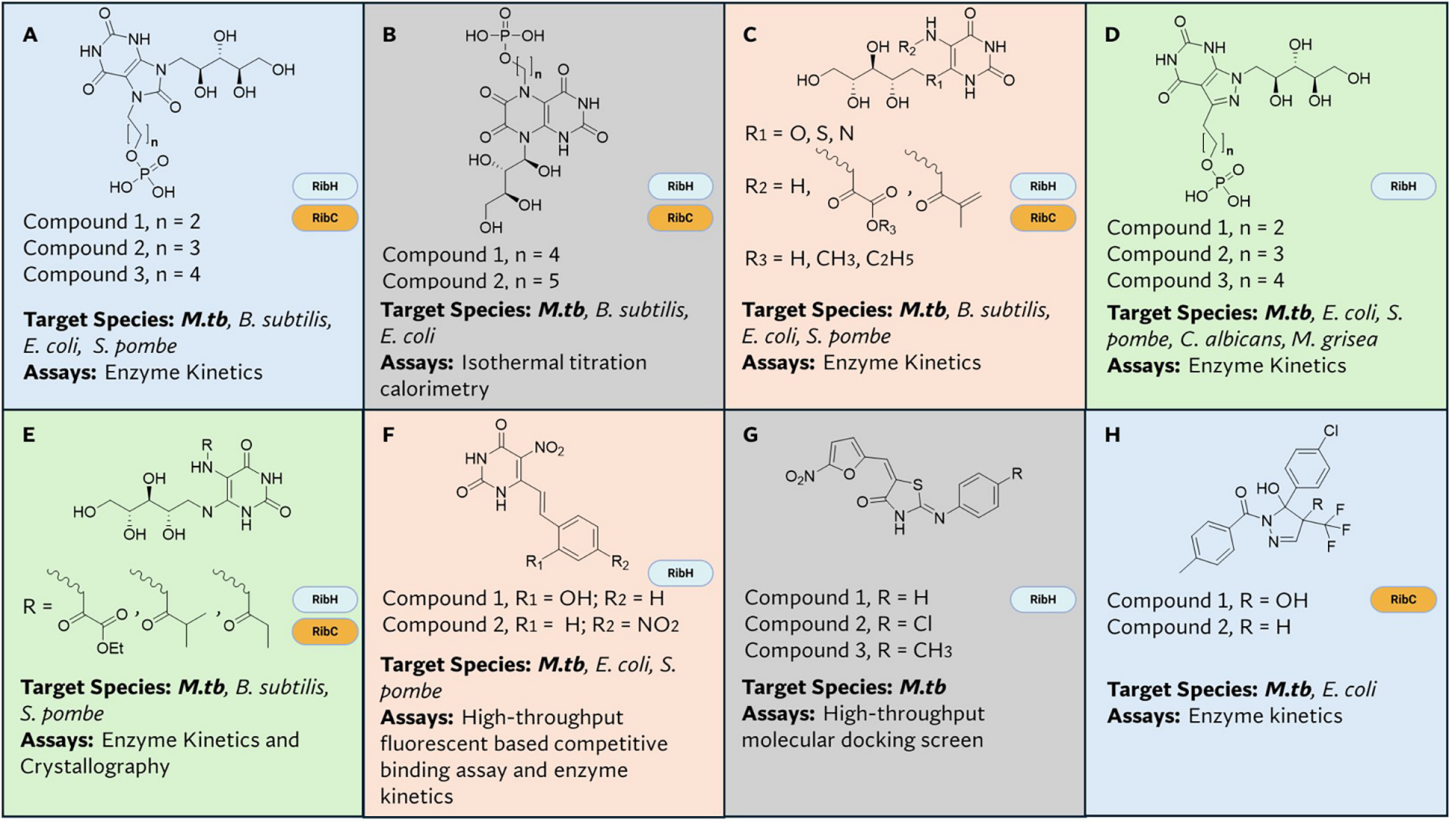
Inhibitors of flavin biosynthesis. This figure summarizes known small-molecule inhibitors of enzymes in the flavin biosynthetic pathway. **(A)** Derivatives of Purinetrione, **(B)** Derivatives of Ribityllumazinedione, **(C)** O, S and N Nucleoside Derivatives of 5-A-RU, **(D)** Derivatives of Pyrazolopyrimidine, **(E)** Oxalamic Acid Derivatives of 5-A-RU, **(F)** Derivatives of 5-A-RU with substituted ribityl side chain, **(G)** Derivatives of Thiazolidin-4-one, **(H)** Derivatives of Trifluoromethylated Pyrazoles. For each molecule, target enzyme(s), target species and method of discovery are indicated.

Efforts to design inhibitors targeting this pathway have largely focused on lumazine synthase (RibH) and riboflavin synthase (RibC), primarily through the development of substrate analogs that mimic their natural ligands (215–223). Following the structural and biochemical characterization of these enzymes in *B. subtilis* and *E. coli* respectively, an early study successfully identified an inhibitory molecule, which, although a weak inhibitor, served as potent scaffold for further optimization (216). Subsequent studies were able to identify molecules, derivatives of ribitylaminolumazines (218) and purinetrione (217), that exhibited improved binding to the lumazine and riboflavin synthase of *E. coli* and *B. subtilis*. These compounds provided a useful scaffold that guided the design of inhibitors against *M.tb*, with three alkyl phosphate (**Figure 4A**) derivatives of purinetrione demonstrating nanomolar inhibition of *M.tb* RibH (224). The resolution of the crystal structure of RibH of *M.tb* catalyzed the structure-based design and the virtual screening campaigns of libraries for enzyme inhibitors (225–227). The binding mode of the three alkyl phosphate derivatives to *M.tb* RibH was then determined in order to optimize the design of better inhibitors (228). Subsequently, two derivatives of ribityllumazinediones (**Figure 4B**) (215) were shown to inhibit the enzymatic activity and bind the RibH of *M.tb* with high affinity, however their bactericidal activity was not characterized. Beyond the ribityllumazine and purinetrione scaffold, additional novel inhibitors of lumazine and riboflavin synthase have been developed. Derivatives of a sulfur nucleoside analogue of the RibH substrate 5-A-RU (**Figure 4C**), inhibited both RibH and RibC of *M.tb (*220). In a follow-up study by the same group, derivatives of O, S, and C nucleoside analogues of 5-A-RU (**Figure 4C**) also demonstrated inhibitory activities against RibH and RibC (221). Further, a series of three 3-alkyl phosphate derivatives of pyrazolopyrimidine analogues (**Figure 4D**) were shown to be potent inhibitors of *M.tb* RibH (223). Another series of oxalamic acid derivatives of 5-A-RU (**Figure 4E**) was shown to bind RibH and RibC of *M.tb* with moderate inhibition (Ki) of these enzymes (222). Using a high throughput screen to identify inhibitors of lumazine synthase in *Schizosaccharomyces pombe*, an analog of 5-A-RU with a substituted ribityl side chain was shown to inhibit *M.tb* RibH (229, 230) (**Figure 4F**).

Although most of these compounds identified as inhibitors of RibH have provided important insights into the binding mode of this enzyme, there has been little success in translation to pharmacological agents, likely due to their inactivation by phosphatases (224) or inability to permeate the mycobacterial cell wall (231). However, recent progress in targeting this pathway has led to the identification of novel inhibitors of RibH in *M.tb.* A recent study using molecular docking of a ~600,000 compound library identified three novel RibH inhibitors with potent antimycobacterial activity and synergy with first-line anti-TB drugs (**Figure 4G**) (227). Interestingly, these compounds possessed unique chemical scaffolds distinct from traditional substrate mimics, marking a significant shift in the design strategy for RibH inhibitors and potentially for inhibitors of other enzymes in this pathway.

There has also been some success in identifying molecules targeting RibC solely. Kaiser et al. (232) designed an enzymatic photometric read out high-throughput screen for inhibitors of specific riboflavin pathway enzymes. Applying this screening format against a commercial library consisting of 100,000 compounds, they identified 127 hits, with two of them (**Figure 4H**) showing significant inhibition of *M.tb* RibC and antimicrobial activity against replicating and non-replicating *M.tb (*233). However, on-target activity of the compounds had not been confirmed. Serer et al. also developed a unique high-throughput screening platform to search for RibC inhibitors against *Brucella* sp (234). They employed a mobility shift assay coupled with a microfluidic system, where inhibition was observed as a reduction in the riboflavin fluorescent signal. Screening a 44,000-compound library led to the identification of 10 inhibitors of which three demonstrated moderate growth inhibition even in the presence of riboflavin (234). However, activity of these molecules against *M.tb* RibC is yet to be determined.

An alternative strategy to inhibit flavin biosynthesis involves targeting the regulation of flavin biosynthesis. Roseoflavin (RoF), a natural riboflavin analog synthesized by *Streptomyces* exhibits antimycobacterial activity (235). RoF can bind to the FMN riboswitch, leading to the downregulation of RBP genes and consequently reducing riboflavin synthesis (236). Another report documented five riboflavin derivatives, modified at the ribityl moiety that displayed moderate antimycobacterial activity (237). Through *in silico* docking, and by demonstrating binding affinity to the FMN riboswitch aptamer of *Lactobacillus plantarum*, FMN riboswitch binding was proposed as one of their mechanisms (237). However, as the presence of an FMN riboswitch in mycobacteria has not been confirmed, the alternative mechanism of RoF and these riboflavin derivatives may explain their antimycobacterial properties. In the case of RoF, it mimics riboflavin and gets converted to inactive cofactors Roseoflavin-5`-monophosphate (RoFMN) and Roseoflavin adenine dinucleotide (RoFAD) which get incorporated into flavoenzymes, either inhibiting or reducing their activity (238). A similar mechanism was also purported for the riboflavin derivatives (237), however, the clinical use of these molecules is impeded by their potential to also disrupt the host flavoproteome. Other inhibitors of riboflavin biosynthesis have also been reported. Ribocil (239), a synthetic small molecule, and 5FDQD (240), a flavin analog, were both shown to be bactericidal to *E. coli* and *Clostridium difficile* respectively via repression of RBP genes by binding to the FMN riboswitch. Similar to RoF, the use of these molecules in mycobacteria is impeded by the lack of an FMN riboswitch. However, an approach of disrupting the regulation of flavin biosynthesis in mycobacteria may be employed for drug targeting.

From the studies highlighted above, it is clear that RibH and RibC are the most widely targeted enzymes in the flavin pathway. This preferential targeting likely stems from the better stability and malleability of their substrates and a better understood mechanism of action than the RibA2 and RibG enzymes (231). Although, several inhibitors for RibH and RibC have been identified, most of these compounds were identified through cell-free assays using recombinant enzymes, hence their efficacy as antimycobacterial agents remains uncertain. *In vitro* and *in vivo* validation is necessary to assess their translational potential; however, such studies have yet to be reported. Also, studies demonstrating the on-target inhibitory effect on the flavin biosynthetic pathway of these molecules will be essential to demonstrating their mechanism of action. With the advent of tools to better understand the structure and function of these enzymes, the discovery of inhibitors of this pathway should be more approachable. Also, allosteric inhibition of these enzymes may serve as an alternative to substrate mimicry which may facilitate targeting other components of this pathway beyond RibH and RibC.

Four major considerations must be addressed collectively when targeting the flavin biosynthetic pathway pharmacologically. First, the relative vulnerability of mycobacteria to inhibition at different steps of the pathway should be evaluated to identify the most effective enzymatic target. For instance, the presence of two functional lumazine synthase homologs in *M.smeg* may complicate pharmacological targeting of this enzyme. It is therefore crucial to assess whether similar functional redundancy exists in other pathogenic mycobacterial species. Second, the ideal target should be the component of the pathway most resistant to mutational escape, thereby minimizing the likelihood of developing antimicrobial resistance. Third, given the pathway’s involvement in the biosynthesis of MR1 ligands, the immunological impact of inhibiting different enzymes on MR1-restricted T cell responses must be considered when selecting a therapeutic target. Finally, the impact of targeting the shared pathway (RibA2, RibG) on the administration of deazaflavin dependent prodrugs must be considered. It is well established that disruption of deazaflavin biosynthesis confers resistance to pretomanid and delamanid (93). Therefore, co-administering pretomanid and delamanid with inhibitors of RibA2 or RibG would be impractical. Collectively, the unique mechanism of action associated with flavin pathway inhibitors offers a promising avenue to circumvent existing drug resistance mechanisms and contribute to sustained therapeutic efficacy.

## 4 Flavin homeostasis and sequestration in mycobacteria

In addition to the substantial energy required to synthesize riboflavin and subsequently FMN and FAD, bacteria must also manage the impact these metabolites have on redox homeostasis. Exposure of reduced flavins to oxygen results in the generation of ROS and flavin radicals, both of which can damage cellular components (241). Therefore, bacteria must tightly regulate flavin biosynthesis and flavin availability to effectively mitigate the potential harm posed by accumulated flavins. Various bacterial species have evolved distinct strategies to address these challenges, including negative feedback regulation mechanisms mediated by riboswitches (101) or the previously mentioned RHOCS (106). Additionally, a highly conserved strategy observed in *Actinobacteria* species, and shared with archaea (242), involves the sequestration of flavins to prevent oxidative stress (104, 105).

To sequester flavins, mycobacteria utilize specialized flavin-sequestering proteins, three of which have been characterized to date. The first is dodecin, a highly conserved protein across mycobacterial species (105). Structurally, dodecin is a homododecameric protein that preferentially binds FMN (243). Unlike typical flavoproteins, which bind flavins as cofactors for enzymatic reactions, dodecin primarily functions in the sequestration and storage of flavins (242). By doing so, it effectively regulates the availability of free flavins within the bacterial cell. The second characterized protein is the flavin-sequestering protein (Fsq), belonging to the FDOR-B protein family, which is also highly conserved among mycobacteria (244). In contrast to dodecin, Fsq preferentially binds FAD. The expression of the *fsq* gene is regulated by the DosRS two-component system, distinguishing it from dodecin (244). Another DosRS-regulated protein potentially involved in flavin sequestration is Acg. Encoded adjacent to the gene for Acr (alpha-crystallin protein), Acg is among the most abundantly expressed proteins during hypoxia-induced dormancy (245). Although classified as a putative nitroreductase based on sequence homology, Acg lacks demonstrable enzymatic activity, leading to the hypothesis that its primary role may be flavin storage, particularly binding FMN (246).

Genetic studies have indicated that dodecin, Fsq, and Acg are non-essential in mycobacteria (244, 247, 248), however investigations into their functions under stress conditions have demonstrated significant roles for these proteins in mitigating stressors during infection. Dodecin abundance increases in response to decreasing oxygen levels in hypoxia-induced dormancy models of *M.tb* and BCG, subsequently returning to baseline levels upon re-exposure to oxygen (249). Additionally, the gene encoding dodecin, *rv1498a*, is upregulated under acidic conditions and nutrient starvation (250). The biochemical characterization of dodecin also showed that the protein had higher affinity for FMN in acidic environment (105). For Fsq, deletion mutants exhibit impaired growth under hypoxic conditions and delayed reentry into active growth phases (244). Similarly, deletion of Acg leads to reduced fitness in both resting and activated macrophages, highlighting its critical role during the establishment of infection (247). Our group has also reported an increase in the abundance of dodecin, Fsq and Acg in response to elevated riboflavin further buttressing their role in flavin homeostasis (251). Beyond these characterized proteins, sequence homology analyses reveal that mycobacteria encode several other related proteins that may also function in flavin sequestration (252). Considering the likely redundancy among these proteins, future studies aimed at distinguishing their individual contributions will provide deeper insights into mycobacterial physiology and fitness.

The role of flavin sequestering in mycobacteria currently points toward the homeostatic control of flavin and potential generation of radicals. However, it will be important to consider other implications for flavin sequestering. For example, could mycobacteria store flavins for “a rainy day” to be used during scarcity of resources? Since during infection, one of the main stressors encountered by mycobacteria is reduced pH, dodecin may play a role in preventing further damage from redox stressors created by excess flavins. Stored flavins may also serve as a source of essential cofactors upon reentry into active growth. Congruently, *fsq* mutants were shown to have an impaired ability to recover from hypoxia induced dormancy (244). Answering such questions will be necessary to optimize targeting this pathway to ensure that upon inhibition of flavin biosynthesis, the bacilli is unable to use stored flavins for baseline functions until the inhibitory agent is withdrawn. Hence, the role of flavin sequestering in mycobacterial physiology must be further elucidated.

## 5 Role of flavin and deazaflavin biosynthesis in MR1 antigenicity and vaccine development

T-cell immunity plays a critical role in controlling mycobacterial infections (253, 254). Beyond the classical CD4- and CD8-restricted T-cell populations, non-classically restricted T-cells, including CD1-restricted (255), γδ (256), and MR1-restricted (257, 258) T-cells, also contribute to antimycobacterial immunity (259). For decades, the antigens recognized by these unconventional T-cells remained elusive, however recent discoveries have revealed that these cells primarily respond to metabolites (57, 255). CD-1 restricted T-cells, particularly Natural killer T (NKT) cells recognize lipids and glycolipids, while the specific ligands for γδ T-cells remain unclear. MR1T cells, including MAIT cells, recognize intermediates and secondary products of the RBP (57, 65, 260, 261). These findings have significantly shifted the paradigm of T-cell antigen recognition. In this section, we cover the immunology, ligandome and vaccine potential of MR1T cells for mycobacterial infection.

### 5.1 MR1 and its immunological significance during mycobacterial infection

MR1 is the most conserved antigen presenting molecule in mammals (262, 263). For instance, the critical antigen binding domains of MR1, the α1 and α2 domains are 90% conserved between mice and humans (263, 264). This high degree of conservation suggests that MR1 plays a fundamental and evolutionarily important function in the immune system that has therefore been maintained by evolutionary pressure (262). MR1T cells were first described in 1993, as a common type of CD4^-^CD8^-^cell expressing an invariant TCR α-chain (TRAV1-2/TRAJ33) (265). These TRAV1–2 expressing cells were later termed MAIT cells given their invariant α-chain, enrichment in mucosal surfaces and later demonstrated to be restricted by MR1 (266). We now recognize that MAIT cells are only a subset of the broader class of MR1T cells. In adults, MAIT cells are the most common MR1T cell and are defined by their expression of an invariant TCR α-chain (TRAV1-2/TRAJ33/20/12 in humans), paired with a limited array of Vβ segments (267, 268), and often express high levels of CD161 (269, 270) and CD26 (271). MR1T cells have been shown to play an important role in microbial defense, providing protection against many different riboflavin producing pathogens, including *Klebsiella pneumoniae (*272), *E. coli (*260), *Francisella tularensis (*273), *Streptococcus pneumoniae (*274), and mycobacterial pathogens (64, 65, 275).

MR1T cells have many features that make them well suited for defense against a wide variety of pathogens. They are enriched in mucosal surfaces, including the lung (276) and gastrointestinal tract (277), the first contact point for most infections. They do not require priming and are therefore able to respond much more rapidly than conventional T cells. They respond with production of critical inflammatory cytokines including IFNγ and TNF, but also with a robust cytotoxic response, more so than conventional T cells and this may be particularly important for intracellular pathogens such as *M.tb (*276). Insights from key studies support the immunological importance of MR1 in *M.tb* defense, including both animal models and human studies. In murine models, MR1 knockout mice exhibit worse control of BCG infection, showing the role of MR1 in mycobacterial defense (275). In non-human primates (NHPs), blocking CD8a to deplete all CD8^+^ cells, including non-conventional T cells like MR1T cells, had a profound impact on bacterial control and dissemination, whereas blocking CD8b to only deplete conventional CD8-positive cells had only a modest effect on lymph node bacterial control (278). Using a similar model, this same group demonstrated that the protection given to NHPs from IV BCG was lost with CD8a depletion, but not CD8b depletion (279). These studies highlight the important role non-conventional CD8-positive T cells, such as MR1T cells, play in control of *M.tb*, but also in vaccine-mediated protection from *M.tb*. Human studies further reinforce MR1’s role in TB defense, with MR1T cells being higher in a Ugandan cohort of TB resisters (280). Additionally, MR1 polymorphisms have been associated with severe and disseminated TB, underscoring the clinical relevance of MR1 in TB pathogenesis (257).

### 5.2 Heterogeneity of MR1 TCR and ligands

**Figure 5:**
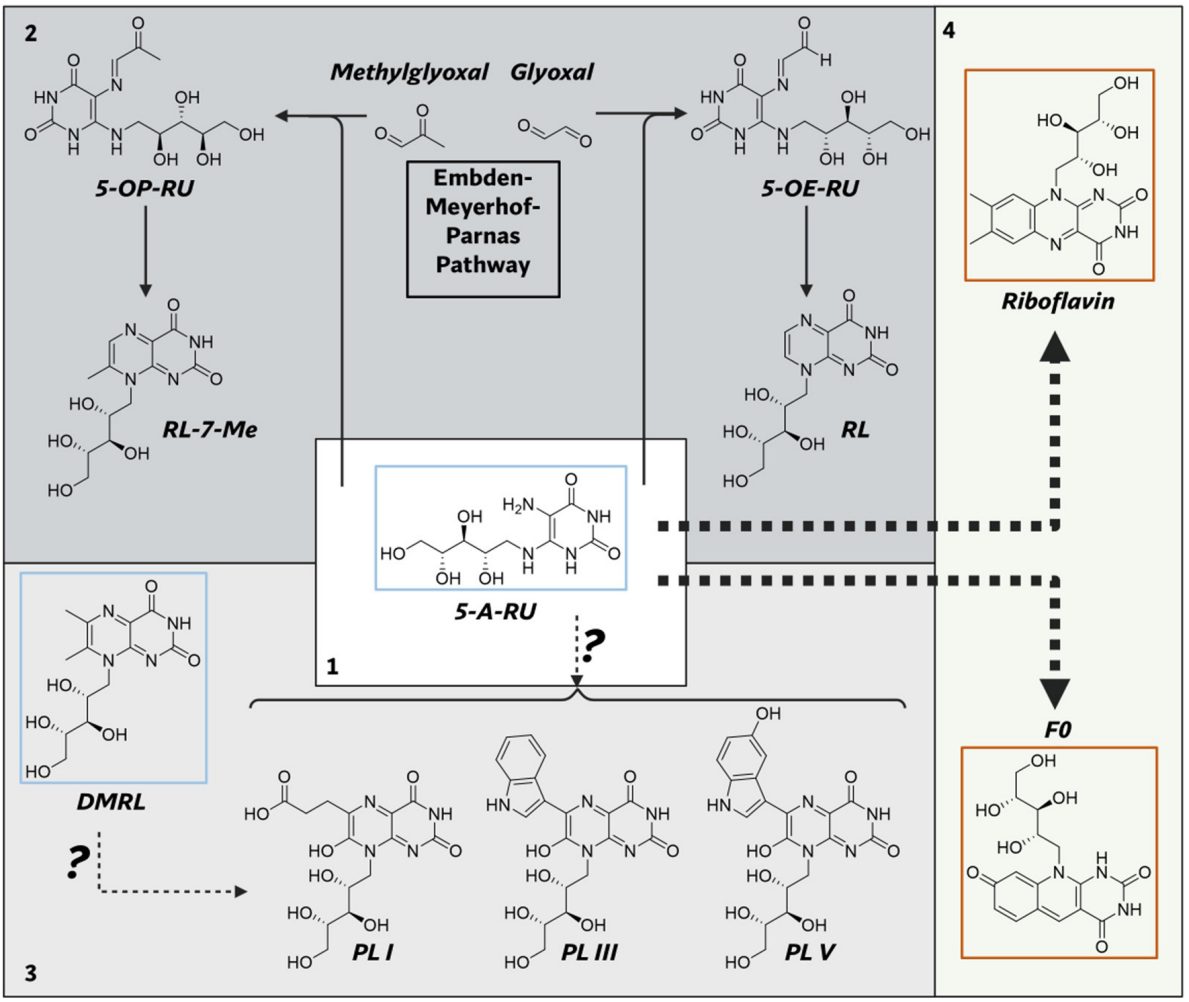
MR1 ligands observed in mycobacteria. MR1 antigens from the flavin and deazaflavin biosynthetic pathway in Mycobacteria. Box 1: 5-A-RU is central to the production of MR1 ligands. Box 2: 5-A-RU reacts non-enzymatically with glyoxal and methylglyoxal, byproducts of glycolysis, to form OE-RU and OP-RU respectively. These products condense to form more stable products: RL and RL-7-Me respectively. Box 3: 5-A-RU serves as a precursor for the synthesis of several MR1 ligands, including DMRL, PL I, PL III, and PL IV. While the PLs have only been observed in M. smeg, their immediate biosynthetic precursor remains unidentified. Box 4: Riboflavin and F0, intermediates of flavin and deazaflavin biosynthesis have been shown as MR1 ligands with antagonistic properties. (Image created with Biorender.com). 5-OP-RU, 5-(-2-oxopropylideneamino)-6-D-ribitylaminouracil; 5-OE-RU, 5-(-2-oxoethylideneamino)-6-D-ribitylaminouracil; RL-7-Me, 7-methyl-8-D-ribityllumazine; RL, ribityllumazine; 5-A-RU, 5-amino-6-D-ribitylaminouracil; DMRL, 6,7-dimethyl-8-ribityllumazine; PL, photolumazine.

Since the initial discovery that intermediates of the RBP serve as ligands for MR1T cells, many other ligands have been discovered, with some functioning as agonists (261, 264) and others as antagonists (87, 258). Among the most potent agonists are 5-OP-RU and 5-OE-RU (**Figure 5**) which bound to majority of the known MR1T cells TCRs (125). This potency was leveraged to develop a MR1-tetramer loaded with 5-OP-RU that allowed researchers to study MR1T cells that do not express canonical markers attributed to MR1T cells (267). The use of MR1-tetramers led to the discovery of TRAV1-2 (–) MR1T cells including MR1T cells that recognized the riboflavin auxotroph, *Streptococcus pyogenes*, in an MR1-dependent manner. These findings started to disprove the hypothesis that the TCR of these T cells were highly invariant. These findings have also spurred investigations to determine the full repertoire of TCRs utilized by MR1T-cells as well as how this impacts the recognition of ligands presented by MR1.

Following the development and expansion of MR1T cells over time has contributed to the observed heterogeneity in their TCR repertoire. Although the majority of MR1T cells in adults are identified as belonging to the subclass of MAIT cells and express the TRAV1–2 chain, this has been shown to differ from what is observed at birth. Using the MR1/5-OP-RU tetramer, the majority of the tetramer(+) cells at birth were identified to be TRAV1-2 (–) (65). Recent work performing TCR sequencing of MR1/5-OP-RU tetramer(+) MR1T cells at birth has further shown that this TCR diversity extends beyond just alternate TRAV usage, with tremendous diversity in TCR usage similar to what is observed in conventional T cells (281). These more diverse cord blood derived MR1T cells were less able to recognize riboflavin producing microbes, suggesting that they may recognize a more diverse array of ligands. Further demonstrating this heterogeneity of MR1T TCRs and the antigens they recognize, work done by Gold et al. showed that different clones of MR1T cells were capable of recognizing riboflavin-producing organisms (*S. typhimirium, C. albicans* and *M.smeg*) in an MR1 dependent manner. However, only some of these clones could recognize the ligand 6,7-dimethyl ribityl lumazine (RL-6,7-diMe) (282). This finding indicates that, even in the context of the ligands derived from the RBP, there is a diversity of ligands.

Similar to the discovery of the diversity of TCR of MR1T cells, understanding of the molecules thought to be presented by MR1 has changed drastically over the years with the discovery of different classes of molecules including folic acid derivatives (57, 125), pyridoxal derivatives (261), nucleobase derivatives (283–285). The diversity of ligands presented by MR1 was further explored by Harriff et al. who used mass spectrometry to examine the *E.coli* and *M.smeg* ligand repertoire presented by MR1 (258). Some ligands were only observed in *M.smeg* and these activated some, but not all, of the MR1T cells clones used in the study. This finding suggests that MR1T cells are capable of discriminating between pathogen-specific ligand repertoires through TCR dependent recognition. Collectively, these studies support the idea that MR1T cells can mount tailored responses to distinct microbial exposures, rather than acting as a uniform invariant population responding to a single conserved antigen. Ultimately, these findings legitimizes the need to identify MR1 ligands, in this case, ligands specific to the mycobacteria genus and species.

### 5.3 The MR1 ligandome of mycobacteria

Since the discovery of 6-formylpterin (5-FP) and 5-OP-RU, the first MR1 antagonist and agonist respectively (57, 125), the list of MR1 ligands has expanded to include both naturally occurring and synthetic molecules. With the recent uncovering of the heterogeneity of TCR utilized by MR1T cells and the binding pocket of MR1 shown to be potentially receptive to other molecules beyond those currently identified, the MR1 ligandome is likely to keep expanding. Here, we will discuss currently identified MR1 ligands in mycobacteria, the current state of research probing the mycobacterial MR1 ligandome and their potential role as immunomodulators.

MR1 ligands are currently classified as antagonist and agonists based on their ability to induce MR1 expression and interact with the TCR of MR1T cells. For both agonists and antagonists, these molecules can be loaded on MR1 in the endoplasmic reticulum (ER) of antigen presenting cells (286, 287), where unloaded MR1 molecules reside, and induce the egress of MR1 to the cell surface where it presents the loaded molecule to the cognate TCR (288). At the point of interaction between the loaded MR1 and the TCR comes the distinction between agonists and antagonists. Agonists induce a response via the TCR activation of MR1T cells leading to cytotoxic activity and the release of proinflammatory cytokines (289). Conversely, antagonists may interact with the TCR but do not induce an immune response (290). This antagonistic property has been shown to prevent TCR dependent activation of MR1T cells via agonists as antagonist loaded MR1 are unable to present agonistic ligands. This competitive inhibition of MR1 agonists presentation by antagonists has been shown experimentally and may play a role in immunomodulation (290). Conversely, the antagonist induced egress of MR1 to the cell surface has also been shown to facilitate the loading of agonist from the extracellular milieu via a yet-to-be-defined exchange mechanism (291). However, it is still unclear how much this exchange mechanism contributes to MR1 antigen presentation during infection. Recently, another mechanism of MR1 antagonism was discovered where antagonists retain MR1 in the ER and prevent its egress to the cell membrane (292). As opposed to antagonists that induce surface expression, antagonists that prevent egress prevent antigen loading both in the ER and the exchange at the plasma membrane. Currently, only synthetic molecules have been shown to utilize the egress-inhibition mechanism. However, the evidence of such mechanism indicates a likelihood of a mycobacteria derived ligand possessing such property.

The FBP and DBP in mycobacteria have been shown to produce both agonistic and antagonistic ligands of MR1 (**Figure 5**). DMRL, is a well-established MR1 agonist, albeit to a lesser extent in comparison to 5-OP-RU (290, 293). F0 and riboflavin have also been shown to be loaded on MR1, although activation studies showed that they had antagonistic properties (258). Beyond these intermediates, photolumazines have also been observed in the environmental *M.smeg*, as previously discussed. Harriff et al. identified Photolumazine I and III using recombinant MR1 in insect cells (258) (**Figure 5**). Krawic and Ladd et al. also identified four isomers of Photolumazine IV in *M.smeg* using a similar system (294) (**Figure 5**). Although none of these ligands have been identified in pathogenic mycobacteria, their discovery provides some context on the expanding family of MR1 ligands in mycobacteria. When these ligands were discovered, they were initially thought to be derivatives of DMRL. However, two recent studies indicate that they are likely derived from 5-A-RU instead. As earlier stated, 5-A-RU was shown to form lumazines, including Photolumazine III, through condensation reactions with transamination products in *E. coli (*126). Also, we recently showed the centrality of 5-A-RU to produce MR1 ligands in mycobacteria. Mutants of *M.smeg* and *M.tb* that lacked RibA2 or RibG were unable to activate MR1T cell clones (295). Interestingly, the loss of DMRL production had little to no impact on MR1T cell activation. Since 5-A-RU itself is not loaded onto the MR1 ligand groove (57), it is hypothesized that 5-A-RU primarily serves as a primer for the synthesis of MR1 ligands. This hypothesis is further corroborated by the ubiquitous nature of 5-A-RU in all riboflavin competent organisms (231). However, 5-A-RU serving as an antigen does not account for the unique MR1 ligandome of different organisms. The findings from these studies, as well as those in the previous section, highlight the need to discover these mycobacterial ligands and understand how they impact MR1 immunology during infection and how the metabolic status of mycobacteria impacts their abundance.

The pathogenic success of mycobacterial species, particularly *M.tb*, partially relies on their capability to modulate the host immune response during infection. The production of MR1 agonists (such as DMRL) and antagonists (such as F0) by mycobacteria highlights their potential role as immunomodulatory agents. Furthermore, during infection of a host, mycobacteria can assume several phenotypically distinct states due to the plasticity of its metabolic landscape (296, 297). This plasticity enables mycobacteria to establish itself as a highly heterogenous population dependent on stressors encountered in host (298–302). These populations differ in cell wall composition, replication dynamics and kinetics, and the status of CCM and accessory pathways. This metabolic plasticity exhibited by mycobacteria enables dynamic alterations in metabolic antigenicity, thus influencing host-pathogen interactions. It is possible that mycobacteria utilize a similar mechanism to modulate surveillance by MR1T cells as its reliance on flavins may change in some of these states. Proteomic analysis of *M.tb* during hypoxia showed a transient increase in the abundance of all the proteins in the riboflavin and deazaflavin pathway except for *fbiB (*249). In the same study, they also observed an increased abundance of FMN and decreased abundance of 5-A-RU and DMRL as oxygen levels depleted (249). It is unclear how these changes may impact MR1 antigenicity as the flux of these intermediates toward downstream products cannot be predicted from this data. Hence, further investigation is necessary to determine how these changes in the proteome and metabolome during hypoxia and other stress-induced dormancy impact the MR1 ligandome of mycobacteria.

It is also clear that the immunological pressure faced by mycobacteria influences its genetic evolution (303, 304). Given the importance of MR1 during mycobacterial infection, it will be important to determine evolutionary changes that might have impacted flavin and deazaflavin biosynthesis in mycobacteria. It was observed that a clinical strain of *Salmonella enterica* had evolved to overexpress *ribB*, the gene that encodes the equivalent of the DHBP synthase activity of *ribA2* in mycobacteria, evaded recognition by MAIT cells (305). Although, it was uncertain how the overexpression of this gene impacted MR1 ligand production, the investigators observed higher levels of extracellular riboflavin and FMN in the *ribB* overexpressor. It is possible that the overproduction of DHBP may drive the pathway toward the synthesis of DMRL and riboflavin and prevent the buildup of 5-A-RU, the required intermediate for MR1 antigens. Similarly, a pathogenic strain of *F. tularensis* was shown to harbor mutations in the *ribA2* gene which negatively impacted MAIT cell activation, a phenotype shown to enhance virulence (306). Another study investigating the impact of overexpressing the *rib* pathway genes in mycobacteria showed increased MAIT activation and decreased virulence in the mouse model when *ribA2* was overexpressed (307). This finding was shown to be MAIT cell dependent as infection of CAST/EiJ mouse model, which has an increased frequency of MAIT cells, led to better protection. Earlier studies in the 1930s attempted, without success, to link riboflavin production with virulence (68). Given recent findings, their original hypothesis may indeed have merit and warrants renewed investigation.

Current research on MR1 ligands in mycobacterial species has predominantly focused on *M.smeg* and *M.tb*. However, the emerging evidence of a broader MR1 ligandome than previously recognized, with species-specific variations, highlights the need to expand ligand characterization efforts to NTMs and *M. leprae*, which exhibit distinct metabolic profiles compared to *M.tb (*8). Growth rate differences between rapid- and slow-growing mycobacteria likely impact the dependence on and the flux of the intermediates through the flavin/deazaflavin biosynthesis. This metabolic divergence suggests pathogen-specific ligand signatures which may influence the role of MR1T cells during infection. With recent advancements in mass spectrometry and bioinformatic tools, the identification of novel natural compounds has become significantly more feasible. It is now an opportune moment to leverage these powerful technologies to systematically identify and characterize MR1 ligands. Discovering both flavin-related and non-flavin-related ligands represents an essential step toward elucidating their roles in MR1-mediated immune responses during mycobacterial infections. Furthermore, pinpointing these ligands will facilitate optimal targeting of MR1-restricted immune cell populations, particularly those residing at the site of infection, enhancing both immunotherapeutic and vaccine development strategies.

### 5.4 Potential of MR1 ligand in vaccines/therapeutics design

**Figure 6:**
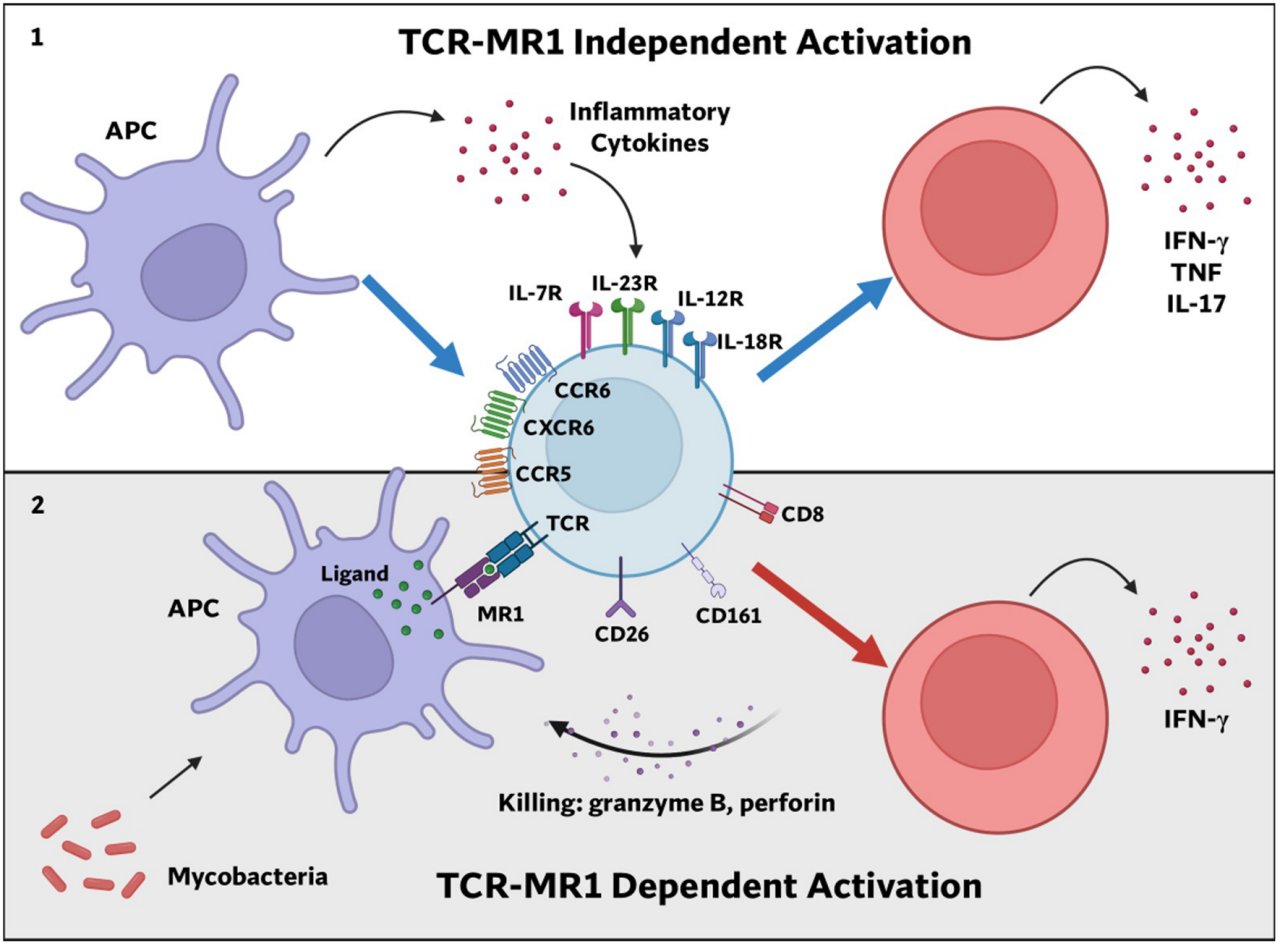
Activation of MR1 restricted T-cells. MR1 restricted T-cells expressing canonical markers of MAIT cells. Box 1: Activation of MR1T cells via a T-cell receptor (TCR) independent mechanism leading to production of pro-inflammatory cytokines. MR1-independent activation is by cytokine stimulation, particularly by inflammatory cytokines such as IL-12 and IL-18. Box 2: Activation of MR1T-cells via a TCR dependent mechanism leading to production of pro-inflammatory cytokines and killing of infected cell. T cell receptor (TCR) activation through MR1, the major histocompatibility complex (MHC)-related protein, is mediated by the recognition of MR1-presented metabolites, predominantly by MAIT cells. MR1 is activated by microbially derived riboflavin intermediates together with co-stimulatory signaling. (Image created with Biorender.com). APC, Antigen Presenting Cell.

MR1T cells have many features that make them attractive vaccine targets. In addition to being enriched in mucosal surfaces (276), the main sites of most initial infections, they are activated more rapidly than conventional T cells, and they have robust cytotoxic capabilities (**Figure 6**) (308). Furthermore, MR1T cells are also active against a broad array of pathogens for which no effective vaccine currently exists. Importantly, unlike the highly polymorphic Major Histocompatibility Complex (MHC), the antigen-presenting molecules that restrict these unconventional T-cells are non-polymorphic (262, 266, 308). This has major implications for vaccine design. Genetic variations in Human Leukocyte Antigen (HLA) genes influence immune responses to vaccination, posing a challenge to developing universal vaccine strategies. In contrast, MR1’s non-polymorphic nature, and the consequent donor-unrestricted characteristics of MR1T cells, suggest that genetic variability is less likely to affect vaccine efficacy.

In mouse models, 5-OP-RU was explored as a vaccine candidate to activate MR1T cells for protection against *M.tb* and while there was robust expansion of MR1T cells in the lung, this was not associated with protection (309). This was also attempted in NHP models, and similarly, 5-OP-RU did not provide protection against *M.tb*, and in fact led MR1T cells to upregulate PD-1 and lose the ability to produce important cytokines such as IFN-γ (310). Although these initial attempts have been unsuccessful, we are only just beginning to understand the complex biology and MR1T cell activation, and utilizing a very broad activating antigen such as 5-OP-RU may not be an appropriate strategy. Work from Riffelmacher et al. (274) has shown that vaccination with the live attenuated *Salmonella* vaccine strain, BRD509, in mouse models led to an expansion of lung-resident antigen adapted MR1T cells with enhanced effector programs. This expansion was associated with a protection against subsequent *Streptococcus pneumoniae* infection, providing proof of concept for an MR1T-based vaccine strategy capable of conferring cross-species protection in mice (274). Additionally, several studies have shown the capacity of MR1T cells to assume an innate memory-like phenotype (273, 276, 277, 311–316). In particular, recent work by Kain et al. showed that following BCG vaccination, there was an expansion of MR1T meta-clonotypes with increased expression of pro-inflammatory and cytotoxic genes, indicative of a recall-like response to prior vaccination (316). Systematically boosted MR1T cell were shown to induce protection against *Francisella tularensis* a month after vaccination with the MR1 agonist, 5-OP-RU (273). Using a similar vaccination strategy, the expansion of lung resident MR1T cells a month prior to infection with *Legionella longbeachae* led to a significant reduction in bacterial burden (315). A recent study showed that *Listeria monocytogenes*, a riboflavin auxotroph, engineered to produce riboflavin conferred protection against *F. tularensis* when used as a vaccine (312). These findings provide evidence of sustainable MR1T cell expansion enabling the development of vaccination strategies targeting MR1T cells. Since most of the studies assessing the vaccine-targeting potential of MR1T cells have evaluated immune response within only a few weeks to a month, further research is required to determine the long-term persistence of the innate, memory-like phenotype of MR1T cells. Understanding the duration and stability of this response will be critical for defining the most effective vaccination strategy that can optimally engage and sustain MR1T cell-mediated immunity.

With the growing recognition of the critical role of MR1T cells during *M.tb* infection, several studies have examined the impact of BCG vaccination on MR1T cell-mediated (316–319). BCG vaccination was shown to modulate the MR1T cell landscape at infancy suggesting its capacity to induce an MR1T cell-mediated response (316). Similarly, BCG revaccination in adults was shown to expand the MR1T cell population, a finding that was recapitulated in non-human primates (317, 319). However, although BCG clearly induces an MR1T cell response, the longevity and the extent to which this response contributes to protection against *M.tb* remains unclear. Furthermore, confirming the similarity between the MR1 ligandome and TCR repertoires elicited by BCG and those triggered by *M.tb* infection is essential to establish effective cross-induction. Finally, identifying the optimal route of BCG administration to maximize the activation of both conventional and unconventional T cells, including MR1T cells, remains an important consideration for vaccine optimization (320).

Although 5-OP-RU broadly activated the majority of MR1T cells, it has been shown that MR1T cells have antigen selectivity (258, 282), and thus the choice of antigen that leads to expansion could play an important role in subsequent protection from infection in a manner akin to conventional T cells. Furthermore, MR1T cells are also cytokine responsive, and activation of MR1T cells in the setting of different cytokine exposure can influence the functional ability of MR1T cells. For example, Wang et al. have recently shown that MR1T cell clones can be induced to switch from IL-17-producing clones to IFNγ-producing clones (311). Thus, it is entirely possible that it is not just the antigen that activates MR1T cells that is important in inducing protection from these cells, but also the cytokine environment that shapes their eventual function and ability to protect against subsequent infection.

Given these facts, identifying MR1 ligands unique to mycobacteria will proffer several advantages in the design of vaccines targeting MR1T cells, Firstly, it will be able to optimize the delivery of the antigens to the right population of immune cells and anatomical region. Secondly, using tetramers loaded with these ligands, it will be feasible to track how different vaccines strategies impact the expansion and function of cognate MR1T cells. Most importantly, the best strategy to ensure the development of memory MR1T cells can be determined. Furthermore, metabolite recognition offers advantages over peptide antigen recognition. Peptide-based immune responses are influenced by post-translational modifications, protein-protein interaction and protein secondary structures, whereas metabolite recognition is comparatively simpler. Additionally, synthesizing and manipulating small molecules is more feasible than working with peptides, making them attractive targets for vaccine development (321, 322).

## 6 Challenges and future directions

**Figure 7:**
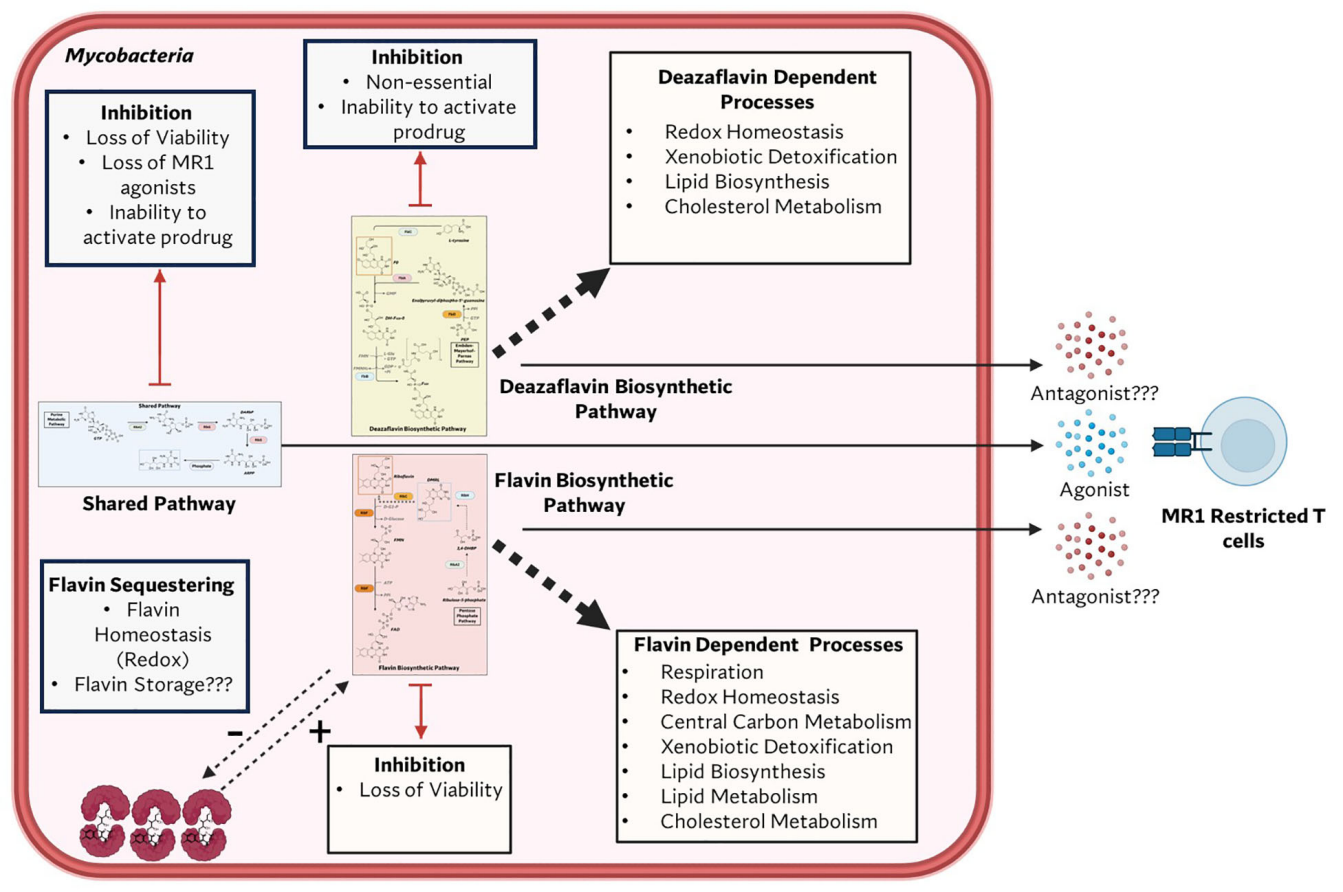
Summary of review. (Image created with Biorender.com).

Flavin and deazaflavin biosynthesis pathways in mycobacteria occupy a unique position at the intersection of antimicrobial discovery and vaccine development, as summarized in **Figure 7**. While deazaflavins contribute significantly to the activation of prodrugs with potent antimycobacterial properties, the rise of resistance mechanisms underscores the urgency to explore novel intervention strategies. Because flavins and deazaflavins are important cofactors for over 160 mycobacterial proteins, most of which remain poorly characterized (107), it is imperative to prioritize functional characterization studies of these flavoproteins and associated enzymes. Although the structural elucidation of many enzymes within these pathways has advanced substantially, critical gaps remain in our understanding of their roles during infection.

In this review, we demarcate FBP and DBP into three components that should be taken into consideration for investigating therapies targeting these pathways. Targeting the shared biosynthetic pathway by disrupting 5-A-RU production, crucial for flavin, deazaflavin, and MR1 antigen synthesis, could profoundly impact MR1-mediated immune responses. It remains essential to assess whether impairing MR1 antigen availability significantly compromises host immunity against mycobacterial infections. Alternatively, inhibiting downstream enzymes, such as those involved in the production of DMRL offers a balanced approach, allowing simultaneous administration of deazaflavin-dependent antimicrobials while preserving MR1 antigen presentation. Additionally, targeting F0 biosynthesis to prevent MR1 antagonism by mycobacteria could further strengthen host immune responses by restricting bacterial immune evasion strategies. Hence, it will be necessary to determine the role of MR1 antagonists in *in vivo* models and determine if blocking F0 or 5-A-RU biosynthesis outweighs the benefit of being able to administer deazaflavin-dependent pro-drugs or the continuous production of MR1 antigens.

Progress in identifying novel MR1 ligands and understanding how structural variations of these molecules influence MR1-restricted T-cell responses is critical. Drawing parallels from other unconventional T-cell systems, such as invariant NKT cells, investigating whether modified MR1 ligands can similarly drive polarized immune responses (pro-inflammatory versus anti-inflammatory) could have profound implications for vaccine design and therapeutic modulation. Moreover, delineating the rules governing cross-reactivity of MR1T-cell receptors, distinguishing responses triggered by exogenous versus endogenous MR1 ligands, and clarifying the physiological significance of extracellular riboflavin represent fundamental questions needing further exploration.

Animal studies employing flavin and deazaflavin biosynthetic mutants, particularly in models that more accurately reflect human MR1T-cell populations, will be instrumental in advancing our understanding of these pathways in granuloma formation and host-pathogen interactions. Given that mycobacteria are unable to scavenge riboflavin or deazaflavin from the host (109), such models will also clarify the impact of targeting these biosynthetic routes on bacterial viability and disease pathology during infection. Additionally, utilizing mutants of flavin-sequestering proteins will provide insights into their functional roles *in vivo*. Moreover, the limited efficacy of 5-OP-RU as a vaccine adjuvant underscores the urgency of identifying physiologically relevant MR1 ligands. These experimental models could be leveraged to explore the contribution of MR1 ligands to the immunological landscape of mycobacterial infection. Ultimately, elucidating how mycobacteria infection modulates host flavoproteins, and how various environmental stressors influence these pathways, will be critical for informing the next generation of antimycobacterial therapeutics and vaccine strategies. Collectively, addressing these research questions holds the potential to unlock novel avenues for targeting flavin and deazaflavin biosynthesis, thereby driving innovation in the development of antimicrobial therapies and vaccines against mycobacterial diseases.

## 7 Conclusion

In this review, we have explored the biosynthetic pathways of flavin and deazaflavin in mycobacteria, highlighting their critical roles in the physiology and immunological interactions of this bacilli. We examined how the products of these pathways contribute to metabolic adaptation, stress resistance, host-pathogen interaction and MR1 antigenicity. Additionally, we discussed the emerging concept of flavin sequestration and its potential role as an integral component of mycobacterial survival. Finally, we outlined key considerations for future research aimed at elucidating these pathways, emphasizing their promise as targets for novel drug development and vaccine strategies. Together, these insights underscore the multifaceted importance of flavin and deazaflavin metabolism in mycobacterial pathogenesis and control.
